# BTBD9-driven IMPDH2 ubiquitination governs sleep homeostasis by gating adenosine metabolism in the basal forebrain

**DOI:** 10.1186/s12964-026-03117-2

**Published:** 2026-07-30

**Authors:** Zhenfei Gao, Niannian Li, Jiawei Feng, Jinhong Shen, Haolin Yuan, Kejia Wu, Xu Xu, Yuenan Liu, Yiming Hu, Shengming Wang, Hui Wang, Xiangshan Yuan, Jian Guan, Feng Liu, Shankai Yin

**Affiliations:** 1 Department of Otolaryngology-Head and Neck Surgery & Center of Sleep Medicine, Otolaryngology Institute of Shanghai Jiao Tong University, Shanghai Jiao Tong University School of Medicine Affiliated Sixth People’s Hospital, Shanghai, 200233 China; 2 Center for Sleep Monitoring and Respiratory Therapy Technology, Institute of Medical Equipment and Technology, Shanghai Jiao Tong University School of Medicine, Shanghai, 200233 China; 3 Department of Otolaryngology & Head and Neck Surgery, School of Medicine, Ruijin Hospital, Shanghai Jiao Tong University, No. 197 Ruijin 2nd Road, Shanghai, 200025 China; 4 Department of Anatomy and Histoembryology, School of Basic Medical Sciences, Fudan University, Shanghai, 200032 China

**Keywords:** Sleep homeostasis, BTBD9, Adenosine, IMPDH2, Ubiquitination, Basal Forebrain

## Abstract

**Background:**

*BTB domain containing 9 (BTBD9)* is among the most reproducible genetic risk factors for sleep-related disorders, yet its molecular function in sleep regulation remains unclear. Here, we aimed to define the downstream substrate, core brain region, and molecular mechanism through which *BTBD9* regulates sleep–wake homeostasis.

**Methods:**

Population-scale genomic and brain expression quantitative trait locus (eQTL) datasets were integrated to infer the direction of *BTBD9*-associated genetic risk in sleep-related traits. Basal forebrain-specific neuronal manipulation of *Btbd9* was combined with electroencephalography/electromyography (EEG/EMG)-based sleep analysis and fiberphotomerty to define the relevant brain locus and functional output. Ubiquitinomics, protein interaction, ubiquitination, and enzymatic assays were used to identify BTBD9-regulated substrates and determine how *BTBD9*-dependent modification affects metabolic enzyme activity. Basal forebrain-specific genetic disruption, receptor pharmacology, and adeno-associated virus (AAV)-delivered interfering peptides were further applied to test pathway necessity and reversibility in vivo.

**Results:**

human genetic analyses showed that *BTBD9* variants associated with higher expression predicted a pro-arousal tendency. Consistently, neuron-specific deletion of *Btbd9* in the mouse basal forebrain increased non-rapid eye movement (NREM) sleep and extracellular adenosine levels. Mechanistically, IMPDH2, the rate-limiting enzyme in de novo purine biosynthesis, was identified as a substrate of the Cullin-RING E3 ubiquitin ligase 3 (CRL3)^BTBD9^ ubiquitin ligase. BTBD9-mediated ubiquitination at IMPDH2 lysine 195 (K195) relieved guanosine triphosphate (GTP)-dependent allosteric inhibition, thereby limiting adenosine accumulation and sleep pressure. Basal forebrain neuronal knockout of *Impdh2* recapitulated the sleep-promoting effect of *Btbd9* deletion, whereas adenosine A1 receptor (A1R) blockade attenuated this phenotype. AAV-delivered interfering peptides targeting the BTBD9–IMPDH2 interaction reduced IMPDH2 ubiquitination, elevated adenosine levels, increased NREM sleep, and rescued BTBD9 overexpression-induced abnormal wakefulness in vivo.

**Conclusions:**

These findings identify a BTBD9–IMPDH2–adenosine pathway that links human genetic risk to basal forebrain control of sleep homeostasis. By defining *BTBD9* as a molecular regulator of purine metabolism, this study provides a mechanistic framework for understanding and potentially targeting *BTBD9*-related sleep dysregulation.

**Supplementary Information:**

The online version contains supplementary material available at https://doi.org/10.1186/s12964-026-03117-2.

## Introduction

Sleep–wake regulation is a fundamental biological process [1], the disruption of which manifests in diverse sleep disorders such as insomnia, obstructive sleep apnea(OSA) related sleep fragmentation, and restless legs syndrome (RLS). Despite their prevalence [2–5], yet the molecular mechanisms governing sleep–wake homeostasis remain poorly understood. Human genetics serves as a powerful entry point for identifying core sleep-regulatory genes and unraveling these intricate molecular frameworks [6]. However, for many risk genes identified through large-scale genomic studies, their precise biological functions and the signaling pathways they govern in the brain remain insufficiently explored [7]. This gap in knowledge significantly hinders our understanding of the pathogenesis of human sleep disorders.

*BTBD9* represents such a genetic factor. Polymorphic variants in *BTBD9* have been consistently associated with multiple sleep-related traits and disorders, including insomnia [8], RLS [9, 10], daytime sleepiness [11], chronotype [12], and sleep fragmentation in OSA [13], suggesting a role for *BTBD9* in human sleep–wake regulation. However, the scarcity of robust expression quantitative trait loci (eQTL) evidence makes it uncertain whether disease-associated SNPs lead to a gain or loss of function, leaving the regulatory direction of *BTBD9* in human sleep homeostasis unresolved.

Furthermore, although early mouse studies suggested that systemic *Btbd9* deficiency contributes to hyperactive movement and sleep disturbance [14–16], interpretation of these phenotypes is complicated by the potential embryonic lethality observed in global *Btbd9* knockout mice [17]. The deviation from Mendelian inheritance implies that surviving adults likely undergo developmental compensation, introducing significant confounding factors that may mask or distort the true homeostatic sleep requirements [17]. In addition, systemic *Btbd9* deletion has been reported to produce divergent electrophysiological effects across different brain regions and neuronal cell types [15, 16]. Notably, even within the striatum, conditional deletion of *Btbd9* produces electrophysiological effects in cholinergic interneurons opposite to those observed following systemic *Btbd9* deletion [16], highlighting the exquisite context-dependency of *BTBD9* in neuronal function. Consequently, the role of *BTBD9* in sleep regulation remains ambiguously defined. Thus, conditional knockout and circuit-specific strategies are imperative to precisely dissect the molecular-to-circuit mechanisms through which *BTBD9* modulates sleep-wake homeostasis.

As a substrate adaptor for the Cullin-3 (CUL3)-based RING E3 ubiquitin ligase complex (CRL3), *BTBD*9’s primary biochemical function is to facilitate the recognition and subsequent ubiquitination of specific target proteins [18]. While the CRL3-mediated ubiquitination system is increasingly recognized as a pivotal regulator of sleep and circadian rhythms [19–21], the neuronal substrates of the CRL3^BTBD9^ complex that mediate its effects on sleep remain unidentified. Leveraging a cell-based quantitative ubiquitinomics approach in our previous work, we identified inosine monophosphate dehydrogenase 2 (IMPDH2) as a high-confidence substrate for BTBD9-mediated ubiquitination [22]. IMPDH2 serves as the rate-limiting enzyme in the *de novo* biosynthesis of guanine nucleotides and sits at a crucial branch point in the purine metabolic pool [23]. Its enzymatic activity is a major determinant of the balance between adenine and guanine derivative levels [24], the former of which directly affect the metabolism of adenosine, a potent endogenous somnogen and a master regulator of sleep-wake homeostasis [25–28]. However, whether this ubiquitination-dependent molecular mechanism operates in vivo to govern neural activity and sleep architecture remains to be established.

In this study, we addressed these long-standing contradictions by integrating population-scale human genomics with circuit-specific mechanistic investigations. By leveraging large-scale genetic and eQTL datasets, we first established the functional polarity of *BTBD9* in human sleep regulation, identifying it as a potent pro-arousal factor. We subsequently demonstrated that BF-specific deletion of *Btbd9* in mice precisely recapitulates this regulatory direction, resulting in a robust increase in NREM sleep. Mechanistically, we identified IMPDH2 as an evolutionarily conserved BTBD9 substrate, and that BTBD9-mediated ubiquitination at K195 acts as a molecular switch that relieves the allosteric inhibition of IMPDH2, thereby gating the purine metabolic flux. We show that this BTBD9-IMPDH2-adenosine axis in the BF serves as a important metabolic gate for sleep-wake homeostasis. Finally, by developing rationally designed interfering peptides to disrupt this interaction, we further demonstrated the functional relevance of this pathway in sleep–wake regulation. Together, our findings bridge the gap between human genetic risk and the molecular-to-circuit control of sleep.

## Matarials and methods

### UK Biobank sleep trait association analysis of *BTBD9* eQTLs

The UK Biobank (UKB) is a large prospective cohort with linked genetic and phenotypic data. Ethical approval was obtained by the UK Biobank Ethics and Governance Council, and all participants provided informed consent. Analyses in this study were performed under UK Biobank application 493,597. All reported *BTBD9* expression quantitative trait loci (eQTLs) were obtained from the GTEx Portal (v10). These SNPs were used as candidate regulatory variants for association analyses with sleep-related traits in the UKB. Sleep traits included sleep duration, chronotype, insomnia symptoms, snoring, and daytime sleepiness, as assessed by baseline touchscreen questionnaire. We then performed SNP–trait association analyses between *BTBD9* eQTL variants and these sleep phenotypes. Association analyses between *BTBD9* eQTL variants and sleep-related traits were performed using linear or logistic regression as appropriate. Given the limited number of candidate SNPs and correlated sleep phenotypes, multiple testing correction was performed using the Benjamini–Hochberg false discovery rate (FDR) method, with q < 0.05 considered statistically significant. Variants showing associations with sleep traits were further filtered to retain those annotated as brain eQTLs for *BTBD9* in GTEx. These brain eQTL SNPs were selected for downstream analysis and visualization in the present study.

### Mice

All procedures were approved by the Institutional Animal Care and Use Committee of Shanghai Jiao Tong University Affiliated Sixth People’s Hospital (no. 2021 − 0149). All mice were housed four to five per cage under a constant temperature (22 ± 0.5 °C), humidity (55 ± 5%), and an automatically controlled 12 h light/12 h dark cycle (lights on at 7 a.m.), with access to food and water ad libitum. C57BL/6J mice were purchased from GemPharmatech; C57BL/6JCya-*Btbd9* KO, C57BL/6J-Cya-*Btbd9*^em1flox^/Cya, C57BL/6J-Cya-*Impdh2*^em1flox^/Cya and C57BL/6J-Cya-*Adora1*^em1flox^/Cya were generated with the assistance from Cyagen. We declared that our study examined male mice because male animals exhibited less variability in phenotype. It is unknown whether the findings are relevant for female mice.

### Cell line

HEK293T, SH-SY5Y and N2a cells were provided by the Cell Bank, Chinese Academy of Sciences, and cultured in Dulbecco’s modified Eagle’s medium (DMEM) plus 10% fetal bovine serum and penicillin and streptomycin at 37 °C in 5% CO_2_ (v/v). The identity of both cell lines was verified by short tandem repeat (STR) profiling.

### Chemicals, viruses, antibodies and other materials

We provided a table in a supplementary file containing all chemicals, viruses, and any other reagent used in this paper, and devices or apparatus as well. Please refer to the Supplementary Table 5.

### Surgical procedure

All surgical procedures were conducted under general anesthesia using continuous isoflurane (5% for induction; 1.5%–2% for maintenance).

### AAV virus injection

For AAV virus injections, 8- to 10-week-old mice weighing 22–25 g are anesthetized and secured in a stereotaxic apparatus. Erythromycin ointment is applied with a cotton swab to keep the eyes moist and prevent light damage. The scalp is trimmed, disinfected with povidone-iodine, and incised, followed by cleaning the exposed bregma and lambda with 1% hydrogen peroxide. Using the bregma as a reference point, a 0.5 mm drill is used to create a hole at AP + 0.1–0.15 mm, ML ± 1.55–1.75 mm, DV -5.4 mm. A microsyringe needle (33 g, 30 mm) is then slowly inserted, and 200–400 nl of virus is injected at a rate of 50 nl/min. After injection, the needle is kept in place for 5 min to prevent virus backflow, then slowly withdrawn. The scalp is sutured, and experiments are conducted after at least two weeks of recovery.

For fiber photometry experiments, different control and Cre viruses are injected into the left and right hemispheres of the same mouse to compare the effects of gene knockout on calcium or extracellular adenosine signals.

### EEG/EMG electrode, optical fiber, and cannula implantation

Following anesthesia, mice were fixed in a stereotaxic frame. For routine sleep recording, two holes were drilled at AP + 1.5, ML -1.5, and AP -3.5, ML + 1.5. A 1 mm diameter, 3 mm length skull screw was implanted approximately 1 mm below the skull surface, with the former serving as the recording electrode and the latter as the reference electrode. The screws were secured to the skull with light-curing resin, and EEG electrodes were wrapped around the screws. For EMG, a silicone-coated EMG electrode was inserted 2 mm into the neck muscles of the mouse. The electrodes were fixed with dental cement. Mice were placed in a cage identical to the recording cage for one-week post-surgery for recovery.

In experiments combining optical fiber and sleep recording, an optical fiber ferrule (200 μm diameter, NA 0.5) was implanted at 0.1 mm above the virus injection site. The EEG recording electrode was placed at AP -3.5 mm, ML -1.5 mm, with the reference electrode above the left cerebellum.

For i.c.v experiments, a guide cannula (O.D 0.47, RWD) was positioned 0.2 mm above the target brain region.

### EEG/EMG recording

Mice for sleep recording were placed individually in a 35 × 25 × 25 cm transparent cage with free access to food and water. The electrodes were connected via a custom-made slip ring to the head stage, and EEG/EMG signals were transmitted through the head stage to the PZA amplifier and processed by the TDT-RZ10X. Data were previewed and recorded online using Synapse (TDT). For EEG signals, the bandpass filter was set at 0.3–30 Hz, and for EMG signals, at 20–200 Hz, with notch filters at 50 Hz, 100 Hz, and 150 Hz. The sampling rate was set to 1017 Hz. Mice were connected to the conductive slip ring by a plunge for at least three days of habituation before signal acquisition. Baseline sleep recordings were averaged over two days, with equal numbers of control and intervention groups in each batch to reduce unknown environmental effects. For experiments requiring simultaneous fiber photometry and EEG/EMG recordings, the formal recording started after a three-hour habituation period.

For EEG/EMG recording after pharmacological interventions by cannula injection. DPCPX (100 nM) and SCH58261 (100 nM) were infused bilaterally into the BF via guide cannula under isoflurane anesthesia at 8:00 PM. Drugs were delivered at a constant rate of 100 nL/min using a microsyringe driven by a pump for a total volume of 1 µL, followed by a 5-minute diffusion period. Mice recovered from anesthesia were returned to their home cages. EEG/EMG recording began about 30 min post-injection to allow full recovery.

### Fiber photometry

The night before fiber photometry, fibers were bleached to minimize autofluorescence. The implanted fiber ferrule was connected to a patch cord via a ceramic sleeve, with 465 nm excitation for GCaMP and adenosine signals and 405 nm for isosbestic control. Excitation intensity was measured at 50–70 µW (465 nm) and 25–30 µW (405 nm) at the fiber tip using a SANWA LP10 power meter. Photometry data was transmitted back to the RZ10X through MiniCube and processed by Synapse, with storage at a sampling rate of 1017 Hz.

### Open field test (OFT)

The open field test was used to assess locomotor activity and anxiety-like behavior. Before the experiment, mice were placed in the experimental environment for one day to adapt to the environment. During the experiment, mice were placed individually into the center of a square open-field arena (40 × 40 × 40 cm) with opaque walls. Their activity was recorded for 10 min using an automated video-tracking system (VisuTrack, CN). Total distance/time traveled and time spent in the central zone (20 × 20 cm) were quantified. The arena was cleaned with 70% ethanol between trials to eliminate olfactory cues. All experiments were performed between 9:00 am to 3:00 pm.

### Novel object recognition (NOR)

The novel object recognition test was performed the day after the OFT test which was equivalent to the Habituation section. Firstly, two identical objects (Object A) were placed in the arena (40 × 40 × 40 cm), and mice were allowed to explore for 10 min, this section was performed as training section. And 1 h later, one familiar object (A) was replaced with a novel object (B), and mice were again allowed to explore for 5 min.

Exploration time and duration were detected and calculated by VisuTrack system.

A recognition index was calculated as: time spent exploring the novel object / total exploration time (duration) × 100%. All objects and arenas were cleaned with 70% ethanol between trials. All experiments were performed between 9:00 am to 3:00 pm.

### Immunohistochemistry (IHC)

Brains were fixed in 4% paraformaldehyde (PFA) for 8 h at room temperature, followed by cryoprotection in 30% sucrose at 4 °C for 1–2 days. Coronal Sects.  (30–50 μm) were cut using a freezing microtome (Leica CM1950). Free-floating sections were permeabilized in PBS with 0.3% Triton X-100 (PBS) and blocked in 5% BSA for 1 h at room temperature. Sections were incubated with primary antibody (anti-NeuN, 1:200; anti-S100β,1:200; anti-IMPDH2, 1:200) at 4 °C overnight, followed by incubation with Alexa Fluor 555/488-conjugated secondary antibody (1:500, Thermofisher, USA) for 2 h at room temperature. Images were acquired using a Zeiss LSM800 confocal microscope.

### Proteomics and metabolomics analysis

Eight-week-old *Btbd9* knockout mice and littermate controls were anesthetized and rapidly perfused with cold phosphate-buffered saline (PBS, 1×) to remove blood. The brains were immediately harvested on ice, and specific brain regions of interest were carefully dissected under a stereomicroscope. Each sample was snap-frozen in liquid nitrogen.

Proteomic experiments, including sample preparation, TMT labeling, LC-MS/MS analysis, and data processing, were performed as previously described [22]. For metabolomic profiling of brain tissue, targeted analysis of nucleotides and inosine monophosphate (IMP) was conducted using UPLC-TQMS (Metabo-Profile, Shanghai, China) on tissue samples collected using the same protocol as proteomics. After methanol-based extraction under chilled conditions, metabolites were analyzed by UPLC-MS/MS. Raw data were processed using MassLynx (v4.1), and quantitative results were calculated using internal standard calibration curves. Multivariate and univariate statistical analyses were conducted using in-house iMAP software. For Adenosine assays with cell line, cells were pre-treated with DMSO or MPA (1µM) for 12 h, then followed by the manufacturer’s protocol (Cell Biolabs, San Diego, USA). All tissue was collected between 11:00 am to 1:00 pm.

### Cell transfection

For transfection, cells were seeded into 6-well plates or 6-cm plates and transfected at 60–80% confluence using Lipofectamine 3000 (Thermo Fisher Scientific, USA) according to the manufacturer’s instructions. A total of 4 µg (6-cm) or 2 µg (6-well) of plasmid DNA was used per transfection. For co-transfection experiments, plasmids were mixed at equal mass ratios unless otherwise indicated. Cells were imaged or harvested 24–48 h post-transfection.

### Dual-luciferase reporter assay

To determine whether rs117733138 affects the transcriptional regulatory activity of the *BTBD9* locus, a dual-luciferase reporter assay was performed. A 301-bp genomic fragment containing rs117733138, including 150 bp upstream and 150 bp downstream of the variant site on the sense strand, was synthesized and cloned into the pGL3-Basic vector. Two constructs were generated: one carrying the wild-type G allele and the other carrying the risk A allele. The empty pGL3-Basic vector was used as control.

Cells were co-transfected with the firefly luciferase reporter plasmids and the Renilla luciferase control plasmid pRL-TK (Promega). Luciferase activities were measured using the Dual-Luciferase Reporter Assay System (Promega) according to the manufacturer’s instructions. Firefly luciferase activity was normalized to Renilla luciferase activity.

### Immunoprecipitation (IP)

HEK293T cells were lysed in IP lysis buffer (Beyotime, CN) supplemented with protease and phosphatase inhibitors (Beyotime, CN). After centrifugation at 12,000 × g for 15 min at 4 °C, the supernatant was collected and incubated with 20 µL of anti-FLAG(DYKDDDK) affinity beads (Selleck, USA) overnight at 4 °C. Beads were washed 3–5 times with lysis buffer, and bound proteins were eluted with SDS sample buffer, boiled, and analyzed by Western blot. All experiments were performed in three independent replicates, with representative images shown.

### Western bolt (WB)

Cells were lysed in RIPA buffer (Beyotime, CN) supplemented with protease and phosphatase inhibitors (Beyotime, CN). Protein concentrations were determined using a BCA assay (Epizyme, CN). Equal amounts of protein (20–30 µg) were separated by SDS-PAGE and transferred to NC membranes (Millipore, USA). Membranes were blocked with 5% non-fat milk in TBS for 1 h at room temperature and incubated overnight at 4 °C with primary antibodies against IMPDH2/FLAG/Myc/HA (1:1000) and GAPDH (1:5000) as a loading control. After washing, membranes were incubated with HRP-conjugated secondary antibodies (1:10000) for 1 h at room temperature. Signals were detected using the Odyssey CLx (Licor, USA) and Image Studio 6.0 (Licor, USA). Band intensities were quantified using ImageJ software. Quantification was conducted using at least three independent biological replicates for statistical analysis.

### Quantitative real-time PCR (qPCR)

Total RNA extraction, reverse transcribed, and quantitative PCR were performed following the manufacturer’s instructions provided by the qPCR kit (EzBioscience, USA). Relative quantitative analysis of the gene expression level was performed in LightCycler 480 II (ROCHE, USA) according to the operating manual.

Gene-specific primers were used as follows:

- *BTBD9* forward: 5’-GGCAACGCTGACAGATGAGAA-3’;
- *BTBD9* reverse: 5’-AGGTAGAATCCTCTAGCTCTGGA-3’;
- *IMPDH2* forward 5’-GCTCCTGTGCCTGATGGAAT-3’;
- *IMPDH2* reverse 5’-CGGGCTCCTCCCCAAAATAA-3’;
- *ACTB* forward: 5’-CATGTACGTTGCTATCCAGGC-3’;
- *ACTB* reverse: 5’-CTCCTTAATGTCACGCACGAT-3’;

Relative mRNA expression levels were calculated using the 2^^−^ΔΔCt method and normalized to the housekeeping gene. Three independent biological replicates were performed to assess the mRNA expreesion.

### Immunocytochemistry (ICC)

SH-SY5Y cells were cultured on confocal dishes and fixed in 4% paraformaldehyde (PFA) for 10 min at room temperature. Cells were permeabilized with 0.2% Triton X-100 in PBS for 10 min and blocked with 5% bovine serum albumin (BSA) in PBS for 30 min. Anti-FLAG (1:500) were diluted in blocking buffer and incubated overnight at 4 °C. After washing with PBS, cells were incubated with appropriate fluorophore-conjugated secondary antibodies (1:500) for 1 h at room temperature in the dark. The confocal dish was counterstained with DAPI (Beyotime, CN) for 5 min and then washed with PBS and imaged using a confocal microscope.

### Adenosine assay

Total protein was extracted from IMPDH2- or IS-transfected HEK293T cells before the assay and quantified using the BCA method. Adenosine levels were then measured in opaque 96-well plates using a commercial assay kit (MET-5090, Cell Biolabs) according to the manufacturer’s instructions. Fluorescence intensity was normalized to total protein content and used to compare adenosine levels between samples.

### Confocal imaging

Live-cell imaging was performed to monitor the expression and membrane localization of the GRAB-ADO sensor. HEK293T cells were cultured on glass-bottom dishes (NEST, CN) and imaged 24–48 h post-transfection. Confocal fluorescence imaging was carried out using a laser scanning confocal microscope (Zeiss LSM 880) equipped with a 488 nm laser for excitation and a 500–550 nm band-pass filter for detecting GRAB-ADO green fluorescence. mCherry signal was collected using a 595/50-nm emission filter to confirm the expression of Interfering Sequence. Solutions containing chemicals or drugs were delivered via a custom-made perfusion system.

### Recombinant protein expression and purification

The coding sequence of *Impdh2*-WT/-K195R was subcloned into a mammalian expression vector with an N-terminal FLAG tag. The plasmids were transiently transfected into HEK293T cells using polyethyleneimine (PEI, Polysciences) according to the manufacturer’s protocol. Cells were cultured with shaking at 37 °C, 8% CO₂ for 72 h. The supernatant was collected by centrifugation and filtered through a 0.22 μm membrane. Recombinant proteins were purified using affinity chromatography with FLAG resin (anti-FLAG M2 beads, Sigma, USA). Eluted proteins were concentrated and buffer-exchanged into PBS using Amicon centrifugal filters (Millipore), and purity was assessed by SDS-PAGE and Coomassie staining.

### IMPDH2 enzyme activity assay

IMPDH2 activity was measured by monitoring NAD⁺-dependent production of NADH, indicated by the increase in absorbance at 340 nm at 32 °C in 96-well microplates. The reaction buffer consisted of 100 mM Tris-HCl, 100 mM KCl, and 2 mM DTT (pH 8.0). The final enzyme concentration was 100 µg/mL, with 0.5 mM NAD⁺, 10 mM ATP, and varying concentrations of IMP (62.5–2000 µM). For GTP inhibition assays, GTP was added at concentrations ranging from 0 to 5000 µM. Free Mg²⁺ was maintained at 1 mM throughout the assay. Reaction rates were fitted to the Michaelis-Menten equation using GraphPad Prism (GraphPad Software, USA) to determine the apparent maximum initial velocity. Normalized *V*max values were calculated as the ratio of velocity in the presence of nucleotides to the *V*max in their absence.

### Negative-staining transmission electron microscopy (TEM)

Purified IMPDH2-WT and IMPDH2-K195R proteins were diluted in enzyme activity buffer and incubated with ATP (final concentration: 100 µM) to assess filament formation. An additional set of samples was supplemented with GTP (final concentration: 500 µM) to evaluate GTP-induced effects. All samples were incubated at room temperature for 30 min before negative staining.

For TEM analysis, 10 µL of each sample was applied to a carbon-coated copper grid. The grids were immediately stained with 1% uranyl acetate for 1 min. Images were acquired using a JEM-1400Plus transmission electron microscope operating at 120 kV.

### Molecular docking

Molecular docking simulations were conducted using the AutoDock Vina software package. Structural preprocessing of both receptors and ligands was performed in AutoDock Tools, with the grid spacing set to 0.375 Å. A genetic algorithm was employed to explore conformational space, with a maximum of 10,000 conformations generated. The docking results were ranked based on binding affinity, and the top-scoring pose was selected for subsequent analysis.

### Molecular dynamics (MD) simulations

All MD simulations were performed with the open-source package GROMACS 2021.5. The AMBER ff14SB force field was applied to parameterize protein structures, and the TIP3P model was used to represent water molecules. Protein-ligand complexes were placed in a cubic simulation box with periodic boundary conditions, ensuring at least 1.0 nm between the protein surface and the box edge. The system topology was generated using the pdb2gmx tool. Energy minimization was performed using the steepest descent method to eliminate unfavorable contacts. Following minimization, the system underwent equilibration in two phases: a 1000-ps position-restrained run under the NVT ensemble (constant number of particles, volume, and temperature), and a 1000-ps run under the NPT ensemble (constant number of particles, pressure, and temperature), with temperature maintained at 300 K and pressure at 1 bar. Subsequently, a 100-ns production MD simulation was carried out using a 2-fs time step. Bond constraints were handled using the LINCS algorithm, and long-range electrostatics were calculated via the Particle Mesh Ewald (PME) method [12]. Structural stability and compactness of the protein were assessed post-simulation by calculating the root mean square deviation (RMSD) and the radius of gyration (Rg) using the gmx rms and gmx gyrate modules in GROMACS.

### Data analysis

#### EEG/EMG data processing and sleep scoring

**Fig. 1:**
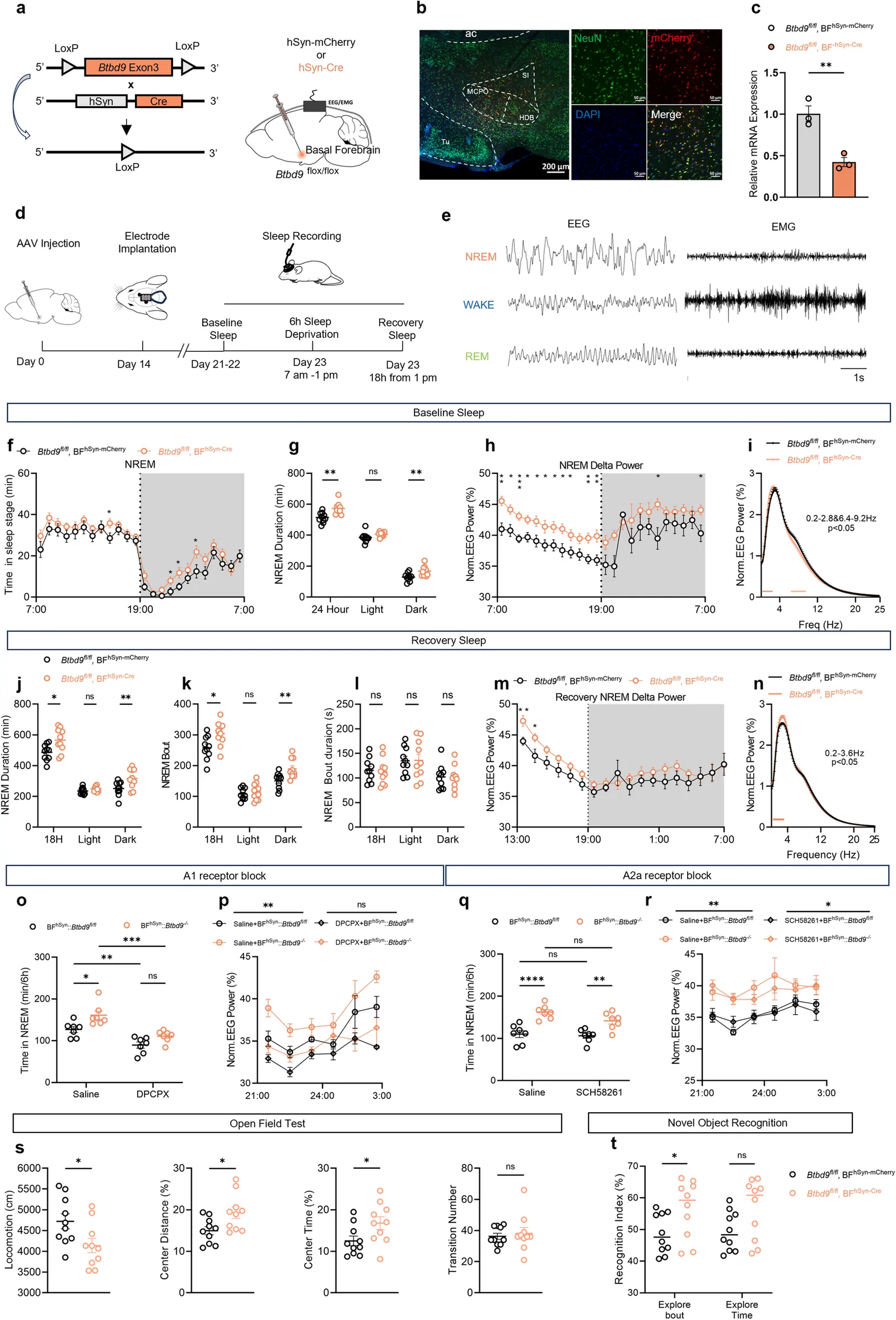
Neuron-specific Btbd9 deletion in basal forebrain enhances sleep via adenosine signaling. **a** Schematic illustration of the generation of Btbd9^fl/fl^ mice and BF neuron–specific Btbd9 knockout (Neu-cKO) mice using AAV-hSyn-Cre. Mice received AAV-hSyn-mCherry or AAV-hSyn-Cre injections in the BF followed by EEG/EMG implantation for sleep recording (*n* = 10 mice per group for baseline sleep recording). **b** Representative images confirming AAV expression in the BF. Scale bar, 200 μm. **c** Validation of Btbd9 knockout efficiency in the BF neurons measured by qPCR (*n* = 3 mice per group). **d** Experimental timeline for sleep recording. **e** Representative EEG and EMG traces corresponding to NREM sleep, wakefulness, and REM sleep used for sleep stage scoring. **f** Hourly time spent in NREM sleep across the baseline recording period (*n* = 10 mice per group for panels **f**-**n**). **g** Quantification of total NREM sleep duration during the 24 h, light phase, and dark phase. **h** Hourly relative EEG delta power during NREM sleep across the baseline recording period. **i** Relative power spectral density of NREM sleep during baseline recording. **j–l** Quantification of NREM sleep parameters during recovery following sleep deprivation, including total NREM duration (**j**), NREM bout number (**k**), and single NREM bout duration (**l**). **m** Hourly relative NREM delta power during recovery sleep. **n** Relative power spectral distribution of NREM sleep during recovery sleep. **o** Total NREM sleep duration after intracranial cannula injection of the A1 receptor antagonist DPCPX from 21:00 to 03:00 (*n* = 7 mice per group for panels **o**-**r**). **p** Hourly NREM delta power following DPCPX injection. **q **Total NREM sleep duration after intracranial injection of the A2a receptor antagonist SCH58261 from 21:00 to 03:00. **r** Hourly NREM delta power following SCH58261 injection. **s** Open field test (OFT) measurements including locomotion distance, center distance, center time, and transition number (*n* = 10 mice per group). **t** Novel object recognition (NOR) performance showing recognition index, exploration bouts, and exploration time (*n* = 10 mice per group). Data are presented as mean ± SEM

For filtered EEG and EMG recordings from mice, sleep-wake states were scored using AccuSleep [29] within MATLAB. Brain states were semi-automatically classified in 5-second epochs and subsequently verified by trained experimenters. Wakefulness was characterized by low-amplitude, high-frequency EEG and elevated EMG activity. NREM sleep was defined by high-amplitude delta waves (0.5–4 Hz) and low EMG activity, while rapid eye movement (REM) sleep showed predominant theta activity (6–10 Hz) with suppressed EMG signals (see Fig. 1e).

#### Sleep power spectrum analysis

EEG signals were subjected to fast Fourier transform (FFT) in 5-second windows aligned with scored sleep states. Power spectral density was computed at 0.2 Hz intervals, and relative power was calculated as the ratio of power within a specified frequency band to the total power. The average power values during WAKE, NREM, and REM periods were determined based on the classified sleep states for each subject.

#### Proteomics and ubiquitinomics data analysis

For both proteomic and ubiquitinomic analyses, candidate proteins and ubiquitination sites were prioritized using a combined nominal significance and effect-size filtering strategy. Student’s t-tests were used for initial group comparisons, and proteins/sites with nominal *P* < 0.05 and a fold-change threshold of ≥ 1.2 were selected as candidate changes for downstream interpretation and validation. Fold changes were calculated as the ratio of group means. Because these omics analyses were used primarily for exploratory candidate prioritization, formal false discovery rate correction was not applied at this screening stage. Functional enrichment analysis was performed as previously described [22].

#### TEM data analysis

To quantify IMPDH2 filament characteristics, transmission electron microscopy (TEM) images were analyzed using ImageJ.

Filament Area Fraction: Filament-like assemblies were manually outlined as regions of interest (ROIs). To account for variations in protein deposition density across different EM fields, the filament area fraction was calculated by dividing the total projected area of filament-like assemblies by the total IMPDH2 particle-positive area (comprising both filamentous structures and non-filamentous components, such as isolated single or free octamer-like particles, while excluding background, artifacts, and damaged regions), expressed as a percentage.

Mean Filament Length: Given that most filament-like assemblies formed relatively straight or near-linear chains, the maximum Feret diameter in ImageJ was used as a 2D estimate for the maximal longitudinal extension of each assembly. The values of all assemblies within a given field were averaged to determine the mean filament length per field.

#### Fiber photometry data analysis

Fiber photometry recordings were acquired at a sampling rate of 1017 Hz for 405 nm (isosbestic) and 470 nm (signal) excitation. The first 10 minutes of data were excluded due to unstable signal fluctuations. Data were subsequently downsampled 1000-fold. GRAB_Ado (or GCaMP) signals were computed by subtracting the 405 nm reference signal from the 470 nm signal. Z-score normalization was applied, followed by baseline drift correction using the “BEADS” MATLAB algorithm (fc = 0.02; amp = 0.00002) and smoothing. Peak detection was performed using the ‘findpeaks’ function in MATLAB with parameters set to ‘MinPeakDistance’ = 10 and ‘MinPeakProminence’ = 2 × SD of the signal.

For simultaneous EEG recordings, sleep state transitions were scored using AccuSleep. We extracted 60-second segments surrounding each transition and used the 10 s immediately preceding the transition as the baseline for further signal analysis.

To assess extracellular adenosine release in vitro, GRAB_Ado-expressing 293T cells were cultured in confocal dishes and treated with genetic or pharmacological interventions. Two hours before the experiment, the culture medium was refreshed. At the start of the recording, baseline fluorescence was captured for 1 min using 488 nm excitation. Then, a custom perfusion system was used to replace the medium with one containing 100 mM KCl to evoke adenosine release. The adenosine signal was quantified as the change in fluorescence (ΔF/F₀), calculated by (stimulated fluorescence-baseline mean) / baseline mean.

#### Statistical analysis

For 24/18/6-hour sleep parameters, statistical analyses were performed according to the structure of the datasets. Total values (e.g., 18-hour NREM duration, total sleep time, and total bout metrics) were compared between two groups using unpaired two-tailed t-tests. For phase-specific analyses (light and dark phases), where measurements were obtained from the same animals across circadian phases, a two-way repeated-measures ANOVA was used with genotype as the between-subject factor and phase (light vs. dark) as the within-subject factor. Šídák’s multiple comparisons test was applied for post-hoc comparisons between genotypes within each phase.

For experiments involving more than two independent groups under a single factor, one-way ANOVA followed by Tukey’s post-hoc test was used.

For time-series data, such as sleep course, PSD course, and PSD spectra, two-way ANOVA was applied with group and time (or frequency) as factors. When repeated measurements were obtained from the same subjects across time points, a two-way repeated-measures ANOVA was performed. Post hoc comparisons were conducted using Fisher’s LSD test.

For validation of gene expression efficiency (e.g., qPCR, Western blot), behavioral assays (NOR, OFT), and sleep parameters measured at a single time point (e.g., NREM duration during the 6 h following cannula drug infusion), unpaired two-tailed Student’s t-tests were used for comparisons between two groups, and one-way ANOVA with Šidák post hoc tests for more than two groups. A paired *t*-test was used for peak comparison in the fiber-photometry test.

Deviation from the expected Mendelian genotype ratio (1:2:1) in offspring from heterozygous intercrosses was assessed using a chi-square goodness-of-fit test. Post hoc 2 × 2 chi-square tests were used to evaluate deviations for individual genotypes.

All data are presented as mean ± SEM. Statistical significance was defined as *p* < 0.05. All analyses were conducted using GraphPad Prism software.

## Results

### Multi-cohort genomic analysis reveals the functional direction of *BTBD9* in human sleep regulation

To define the directional role of *BTBD9* in human sleep–wake regulation, we employed a three-pronged genetic approach. First, we sought to determine how the previously identified sleep-disorder-associated variants influence brain *BTBD9* expression. By interrogating published sleep-related GWAS loci using GTEx data (Supplementary Tables 1 & Supplementary Table 2), we identified rs6922128 as the only sleep-associated variant that also showed detectable BTBD9 eQTL evidence in brain tissues [30], which is significantly associated with decreased *BTBD9* expression in the brain (Fig. S1a & Supplementary Table 3), suggesting that lower *BTBD9* levels may confer protection against insomnia.

Second, we performed a reciprocal analysis to assess whether SNPs with established brain eQTL effects on *BTBD9* correlate with population-scale sleep phenotypes. Systematic analysis of 404,522 participants from the UK Biobank (UKB) database across six independent sleep traits further revealed that 144 brain eQTL loci associated with daytime sleepiness (Supplementary Table 4), and 97/144 loci linked to increased *BTBD9* expression significantly correlated with reduced daytime sleepiness, whereas 19/144 loci linked to decreased *BTBD9* expression showed the opposite trend (Fig. S1b).

Third, we experimentally validated the regulatory potential of a risk variant identified in our previous OSA cohort [13]. Dual-luciferase reporter assays demonstrated that rs117733138 (G > A), a variant strongly associated with abnormal arousal, acts as a functional transcriptional enhancer for *BTBD9* expression (Fig. S1c). Collectively, these converging lines of human genetic and experimental evidence demonstrate that increased BTBD9 expression is associated with a greater propensity for arousal.

### Neuron-specific *Btbd9* deletion in basal forebrain enhances sleep via adenosine signaling

Given the potential limitations associated with global *Btbd9* deficiency [17], we employed circuit-specific manipulations to dissect its role in sleep regulation. To explore the potential brain regions involved in BTBD9-mediated sleep regulation, we analyzed its spatial expression using the Allen Brain Atlas [31], which revealed enriched *BTBD9* expression in the cortex, hippocampus, basal forebrain and basal ganglia (Fig. S2a). Given that the BF is a central hub for sleep homeostasis and is highly sensitive to adenosinergic/purinergic signaling [26, 32–34], a pathway we previously linked to *BTBD9* function through the cellular ubiquitinomic analysis [22] we prioritized the BF as a primary candidate region for functional investigation.

Following the confirmation of *Btbd9* mRNA expression in both neurons and astrocytes of the mouse BF (Fig. S2b-d), we next investigated its cell-type-specific roles in sleep regulation. To this end, we generated BF neuron-specific (Neu-cKO) (Fig. 1a-c) or astrocyte-specific (As-cKO) (Fig. S4a-c) *Btbd9* knockout mice by bilaterally injecting AAV-h*Syn*-Cre or AAV-*GfaABC1D*-Cre into the BF of *Btbd9*^fl/fl^ mice, respectively, followed by comprehensive EEG/EMG recording and sleep stage scoring (Fig. 1d, e). Notably, consistent with the directional role of *BTBD9* in human sleep regulation inferred from our genetic analysis, the BF Neu-cKO mice exhibited a significant increase in NREM sleep duration, especially during the active dark phase (Fig. 1f, g), accompanying elevated NREM bout (Fig. S3a) and enhanced NREM slow-wave activity (SWA) (Fig. 1h, i), a well-established electrophysiological marker of elevated sleep pressure [35]. Consistently, wake duration was significantly reduced, with more WAKE bout and shorter mean WAKE bout duration (Fig. S3c-f), while the total REM sleep duration remains unchanged but shows fragmentation in the knockout group (Fig. S3g-j). Sleep deprivation experiments further revealed an increased recovery NREM sleep duration in the knockouts (Fig. 1j), which was characterized by more NREM bouts (Fig. 1k) without changes in average bout duration (Fig. 1l). In addition, elevated SWA was also observed during early recovery sleep (Fig. 1m, n), further indicating an increase in homeostatic sleep pressure. In sharp contrast, BF As-cKO of *Btbd9* did not alter either baseline sleep architecture (Fig. S4) or recovery responses following sleep deprivation (Fig. S5), suggesting that the sleep-regulation effects of *BTBD9* are mediated specifically through neuronal, rather than astrocytic mechanisms in the BF.

To test whether *BTBD9* regulates sleep through adenosine signaling in the BF, we locally administered adenosine receptor antagonists in BF Neu-cKO mice. Administration of the A1R antagonist DPCPX [36], but not the A2aR antagonist SCH58261 [37], reversed the NREM promoting effect during the early dark phase (21:00–03:00) and SWA, when baseline NREM sleep differences are most pronounced (Fig. 1o–r). These findings are consistent with the involvement of A1R-dependent signaling in the BF in the sleep phenotype associated with *Btbd9* deletion.

Adenosine signaling is known to regulate both learning and memory processes as well as emotional behaviors [38, 39]. Therefore, we performed the novel object recognition (NOR) test and the open field test (OFT) to assess recognition memory and anxiety-like behavior. In the OFT, the *Btbd9* BF Neu-cKO mice exhibited a mild reduction in anxiety-like behavior, evidenced by increased time and distance in the center zone, alongside reduced total locomotor activity (Fig. 1s). Meanwhile, the NOR test revealed that the cKO mice exhibited better cognitive performance than their wild-type controls (Fig. 1t). Given the established role of sleep in cognitive function, these behavioral changes are consistent with the increased NREM sleep observed following BF-specific *Btbd9* deletion.

### IMPDH2 is a preferable target of CRL3^BTBD9^ E3 ligase

**Fig. 2:**
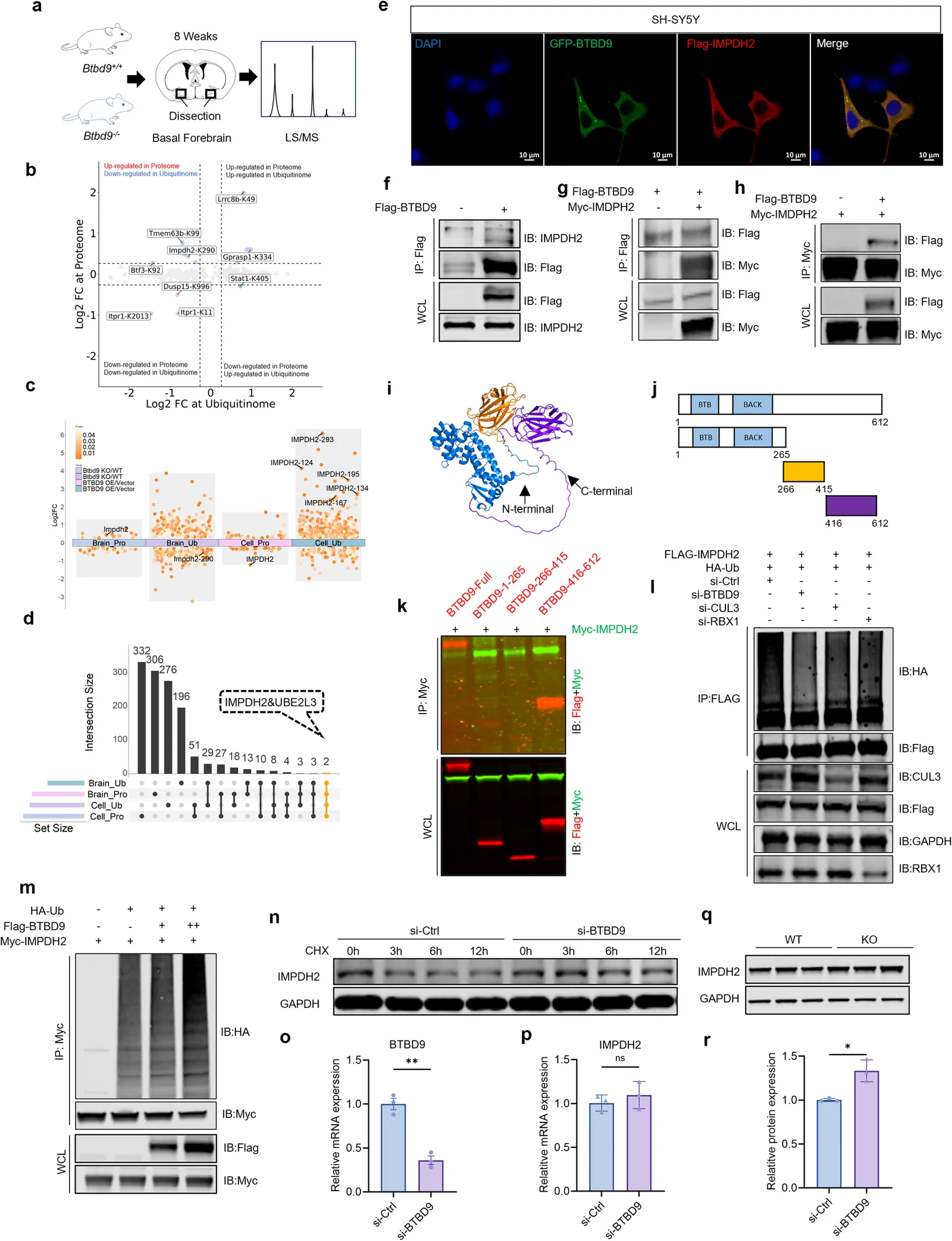
Identification of IMPDH2 as a downstream target of BTBD9. **a** Experimental workflow for proteomic and ubiquitinomic analyses of BF tissues from wild-type and Btbd9 knockout mice. **b** Scatter plot comparing changes in protein abundance and ubiquitination levels in basal forebrain tissues from Btbd9 knockout mice. Each point represents a ubiquitinated site identified in the brain ubiquitinome dataset (*P* < 0.05, fold-change not restricted). Proteins showing increased abundance but reduced ubiquitination in Btbd9 knockout mice were considered preferential candidate targets of BTBD9-mediated ubiquitination (left and up). **c** Integrated volcano-style plot showing results from four datasets (*P* < 0.05 in each dataset): brain proteome (Brain_Pro) and brain ubiquitinome (Brain_Ub) derived from BF tissues of Btbd9 knockout mice, together with previously generated proteomic (Cell_Pro) and ubiquitinomic (Cell_Ub) datasets from BTBD9 overexpression experiments in cultured cells. Proteins showing opposite regulatory directions between proteome and ubiquitinome within either the brain datasets or the cell datasets were considered preferential candidate targets of BTBD9-mediated ubiquitination. Representative IMPDH2 ubiquitination sites are labeled. **d** UpSet plot showing the overlap of significantly regulated proteins across the four datasets (Brain_Pro, Brain_Ub, Cell_Pro, and Cell_Ub). Brain_Pro indicates the proteome of Btbd9 knockout basal forebrain tissue, and Brain_Ub indicates the ubiquitinome of the same tissue. Cell_Pro and Cell_Ub correspond to proteomic and ubiquitinomic datasets from BTBD9 overexpression experiments in cultured cells. The intersection highlights IMPDH2 and UBE2L3 as shared candidates across datasets. **e** Confocal microscopy images of SH-SY5Y cells co-expressing GFP-BTBD9 and FLAG-IMPDH2. Scale bar, 10 μm. **f** Co-IP analysis showing interaction between FLAG-BTBD9 and endogenous IMPDH2 in HEK293T cells overexpressing FLAG-BTBD9. **g–h** Reciprocal Co-IP assay of HEK293T cells co-expressing FLAG-BTBD9 and Myc-IMPDH2 confirming the interaction between BTBD9 and IMPDH2. **i** Predicted three-dimensional structure of BTBD9 generated by AlphaFold3. **j** Schematic illustration of BTBD9 truncation mutants used for interaction mapping. **k** Co-IP assay showing the interaction between Myc-IMPDH2 and FLAG-tagged BTBD9 deletion mutants. **l** Ubiquitination assay in HEK293T cells expressing HA-Ub and FLAG-IMPDH2 with siRNA targeting BTBD9, CUL3, or RBX1. **m** Ubiquitination assay in HEK293T cells expressing HA-Ub, Myc-IMPDH2, and increasing levels of FLAG-BTBD9. **n** Cycloheximide (CHX) chase assay examining IMPDH2 protein stability in HEK293T cells transfected with siRNA targeting BTBD9 or control siRNA. **o**-**p** Quantitative RT-PCR analysis of BTBD9 (o) and IMPDH2 (p) mRNA levels in HEK293T cells transfected with control or BTBD9 siRNA (*n* = 3 per group). **q**-**r** Immunoblot and quantitative analysis of endogenous IMPDH2 protein levels in brain tissues from wild-type and Btbd9 knockout mice (*n* = 3 per group). Data are presented as mean ± SEM

To identify the direct molecular targets through which *BTBD9* governs adenosine signaling in a physiological context, we performed an unbiased, integrated quantitative proteomic and ubiquitinomic analysis specifically using BF tissues from *Btbd9* knockouts (Fig. 2a). While our previous cellular *BTBD9* overexpression models provided initial candidates [22], this in vivo profiling in the BF was essential to pinpoint the substrates that truly operate within the brain’s complex metabolic landscape and to avoid potential artifacts of overexpression.

These analyses from BF tissue revealed that *Btbd9* deletion leads to widespread alterations in protein ubiquitination, with both increases and decreases across distinct substrate*s* (Fig. S6a, b), whereas only modest changes in protein abundance were observed (Fig. S6c, d), suggesting that BTBD9 may regulate substrates primarily through non-degradative ubiquitination. We observed that several genes previously associated with neurodegenerative conditions, including SCN2A [40], also exhibited altered ubiquitination. Nevertheless, when prioritizing candidates based on cross-species conservation and concordant changes across both proteomic and ubiquitinomic datasets by cross-referencing these in vivo datasets with our previous candidate list, IMPDH2, the rate-limiting enzyme in purine metabolism, emerged once again as the one of the most robust and evolutionarily conserved substrates (Fig. 2c, d).

To characterize the molecular association between BTBD9 and IMPDH2, we first performed immunostaining in SH-SY5Y cells and the confocal imaging revealed that both BTBD9 and IMPDH2 exhibit a broad and overlapping distribution throughout the cytoplasm, without signs of mutual exclusion (Fig. 2e). This spatial proximity prompted us to examine their physical association. Indeed, BTBD9 was found to physically interact with IMPDH2. Both endogenous and overexpressed Co-IP assays demonstrated a robust and reciprocal association between these two proteins (Fig. 2f-h). To identify the minimal binding interface, we utilized *AlphaFold3*-predicted structural models to design a series of BTBD9 truncation mutants. Subsequent Co-IP assays successfully mapped the IMPDH2-interacting domain to the residues 416–612 of BTBD9 (Fig. 2i-k). Given the role of BTBD9 as a substrate adaptor of the CRL3 E3 ligase complex, which comprises CUL3 and RBX1, we examined whether IMPDH2 ubiquitination depends on this machinery. Silencing *RBX1*, *CUL3*, or *BTBD9* using small interfering RNAs (siRNA) markedly reduced IMPDH2 ubiquitination (Fig. 2l). Conversely, *BTBD9* overexpression significantly enhanced IMPDH2 ubiquitination (Fig. 2m). To examine whether BTBD9-mediated ubiquitination affects IMPDH2 stability, we assessed its protein half-life using cycloheximide (CHX) chase assays, and revealed that depletion of *BTBD9* prolonged the protein half-life of IMPDH2 (Fig. 2n) without affecting mRNA levels (Fig. 2o, p). Consistently, we confirmed a slightly increased IMPDH2 protein abundance of the BF tissue from *Btbd9* knockout mice (Fig. 2q, r). Collectively, our current data, together with our previously published in vitro ubiquitination assays [22], establish IMPDH2 as a physiological substrate of BTBD9 mediated ubiquitination.

### BTBD9 enhances IMPDH2 enzymatic activity and regulates adenosine levels

**Fig. 3:**
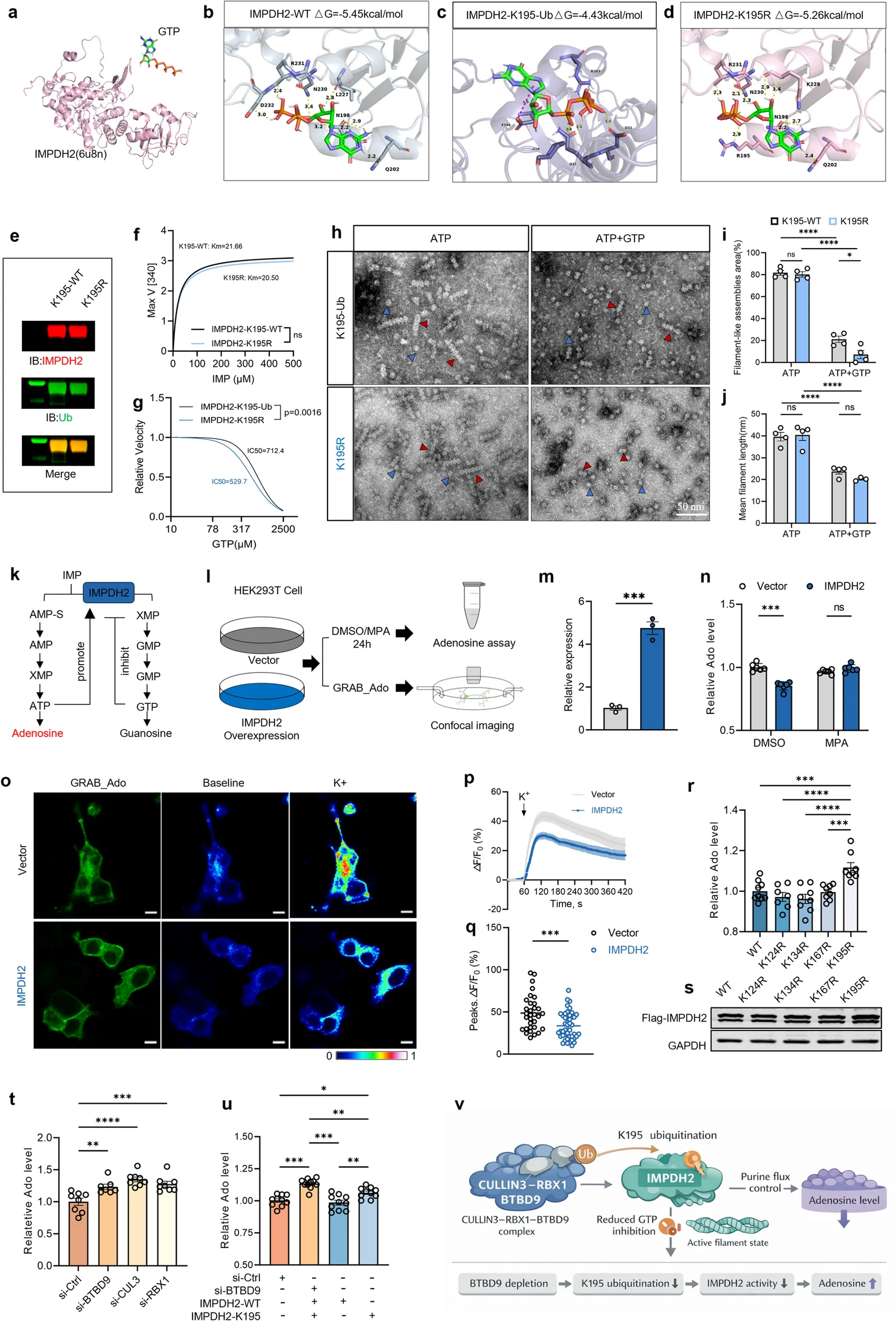
BTBD9 enhances IMPDH2 activity to control adenosine levels. **a** Structural model of IMPDH2(PDB: 6ubn) bound to GTP used for molecular docking simulations. **b**-**d** AutoDock docking results showing the predicted binding of GTP to wild-type IMPDH2 (**b**), ubiquitinated IMPDH2-K195-Ub (**c**), and the IMPDH2-K195R mutant **d**. **e** Western blot detection of ubiquitination of purified IMPDH2 proteins. **f** Enzymatic activity of purified wild-type IMPDH2 and the K195R mutant across increasing concentrations of the substrate IMP. Higher maximal velocity indicates increased catalytic activity of the enzyme. **g** Inhibition of IMPDH2 enzymatic activity by increasing concentrations of GTP in wild-type and K195R proteins. A stronger inhibitory effect reflects greater sensitivity of the enzyme to GTP-mediated feedback regulation. **h** Negative-stain electron microscopy images of wild-type IMPDH2 and the IMPDH2-K195R mutant in the presence of ATP or ATP plus GTP. IMPDH2 can assemble into filament-like structures, which are generally associated with a more active enzymatic state. ATP promotes filament assembly, whereas GTP suppresses filament formation. Blue arrows indicate representative isolated particles, and red arrows highlight filament-like assemblies. Scale bar, 50 nm. **i** Quantification of filament-like assembly area in IMPDH2-K195-WT and K195R under ATP and ATP + GTP conditions. **j.** Quantification of mean filament length of individual filament-like assemblies under the same conditions. **k **Schematic illustration of the role of IMPDH2 in purine metabolism linking guanine nucleotide synthesis to adenosine production. **l** Experimental design of the cellular adenosine assay using HEK293T cells expressing vector control or IMPDH2. **m** qRT–PCR validation of IMPDH2 overexpression (*n* = 3 per group). **n** Intracellular adenosine levels in HEK293T cells expressing IMPDH2 or vector control (vector *n* = 6, IMPDH2 *n* = 7). **o**-**p** Representative GRAB_Ado fluorescence images, scale bar, 10 μm (**o**) and averaged traces (**p**) showing adenosine release following KCl stimulation in vector control and IMPDH2-overexpressing cells. **q** Quantification of peak GRAB_Ado responses following KCl stimulation (vector *n* = 35 ROIs, IMPDH2 *n* = 41 ROIs). **r **Adenosine levels measured in HEK293T cells expressing IMPDH2 mutants at different ubiquitination sites, highlighting the effect of the K195R mutation. **s** Western blotting analysis of IMPDH2 mutant expression. **t** Adenosine levels measured following knockdown of BTBD9, CUL3, or RBX1 in HEK293T cells. **u** Adenosine levels in HEK293T cells expressing wild-type IMPDH2 or the IMPDH2-K195R mutant following BTBD9 knockdown. **v** Proposed model in which the CUL3-RBX1-BTBD9 complex promotes IMPDH2 K195 ubiquitination to regulate purine flux and adenosine levels. Data are presented as mean ± SEM

Since *BTBD9* depletion caused only modest changes in IMPDH2 protein abundance, we hypothesized that BTBD9-mediated ubiquitination instead modulates IMPDH2 enzymatic activity to influence sleep. IMPDH2 activity is tightly controlled by GTP-mediated allosteric feedback inhibition, a critical mechanism for maintaining purine homeostasis [41]. Building on our identification of four potential ubiquitination sites (K124, K134, K167, and K195), our structural analysis revealed that K195 is strategically and uniquely positioned within this allosteric GTP-binding pocket. We therefore hypothesized that BTBD9-mediated ubiquitination at K195 might mechanistically interfere with this feedback loop, thereby modulating purine flux [24] (Fig. 3a, b). Molecular docking revealed that ubiquitination at K195 (by molecular dynamic simulation, Fig. S7) reduced GTP binding affinity (ΔG: -5.45 to -4.43 kcal/mol; Fig. 3c, S8c), while the K195R mutation showed no such effect (Fig. 3d, S8b). To empirically test this regulatory mechanism, we next purified FLAG tagged IMPDH2 protein from mammalian cells to preserve their native post-translational ubiquitination status (Fig. 3e, f) and performed enzymatic assays. While the K195R mutation, which prevents ubiquitination at this site, did not change the maximal catalytic capacity of IMPDH2 (*V*max) (Fig. 3g), but made the enzyme more susceptible to inhibition by GTP, indicating that IMPDH2 activity is more readily suppressed (Fig. 3h). Critically, IMPDH2 activity is structurally coupled to its assembly into high-order filaments, a high-activity conformation promoted by ATP, whereas GTP binding antagonizes filament assembly and promotes disassembly, resulting in enzymatic inhibition. To test whether K195 ubiquitination influences this structural transition, we performed negative-stain electron microscope (EM), which revealed that the less ubiquitinated K195R mutation enhanced GTP-induced filament disassembly, indicating a shift toward a less active enzymatic state (Fig. 3i). Together, these findings demonstrate that ubiquitination at K195 relieves GTP-dependent allosteric inhibition of IMPDH2 activity.

These findings prompted us to examine whether K195 ubiquitination of IMPDH2 by BTBD9 affects adenine metabolism in cells (Fig. 3j). We first examined whether IMPDH2 alone influences cellular adenosine levels (Fig. 3k). Overexpression of IMPDH2 in HEK293T cells significantly reduced intracellular adenosine levels (Fig. 3l), and this effect was reversed by 1 µM mycophenolic acid (MPA), a non-selective IMPDH inhibitor (Fig. 3m), indicating that IMPDH2 regulates intracellular adenosine through its enzymatic activity. To assess whether IMPDH2 also affects adenosine release, we monitored extracellular adenosine using the genetically encoded sensor GRAB_Ado under high K⁺ stimulation [30] and found that *IMPDH2* overexpression reduced extracellular adenosine levels under stimulation (Fig. 3n–p). We next examined the role of K195 ubiquitination on adenine metabolism. Compared with the wild-type construct, the K195R mutant exhibited significantly increased adenosine levels, suggesting that the ubiquitination at K195 is essential for the decrease of adenosine levels by IMPDH2 overexpression (Fig. 3q). Importantly, protein expression levels were comparable between constructs (Fig. 3r), indicating that the effect was not attributable to differences in IMPDH2 abundance.

To further determine whether BTBD9 regulates adenosine metabolism through IMPDH2 in a K195-dependent manner, we performed a functional rescue experiment under *BTBD9* depletion. We first examined the effect of CRL^BTBD9^ depletion on cellular adenosine levels. Knockdown of *BTBD9* and other CRL^BTBD9^ components significantly increased intracellular adenosine, establishing that loss of *BTBD9* is sufficient to perturb adenosine homeostasis (Fig. 3s). To test whether this metabolic control requires K195-specific regulation, we overexpressed either wild-type *IMPDH2* or the K195R mutant in *BTBD9*-depleted cells. Notably, wild-type IMPDH2 significantly attenuated the adenosine elevation caused by *BTBD9* loss, whereas the ubiquitination-deficient K195R mutant showed markedly reduced rescue efficiency (Fig. 3t). Together, these findings demonstrate that BTBD9-mediated ubiquitination of IMPDH2 at K195 modulates IMPDH2 enzymatic activity and cellular adenosine metabolism, suggesting a mechanistic pathway through which BTBD9 may influence adenosine-dependent sleep regulation (Fig. 3u).

### Basal forebrain IMPDH2 controls sleep-wake homeostasis via local adenosine mechanisms

**Fig. 4:**
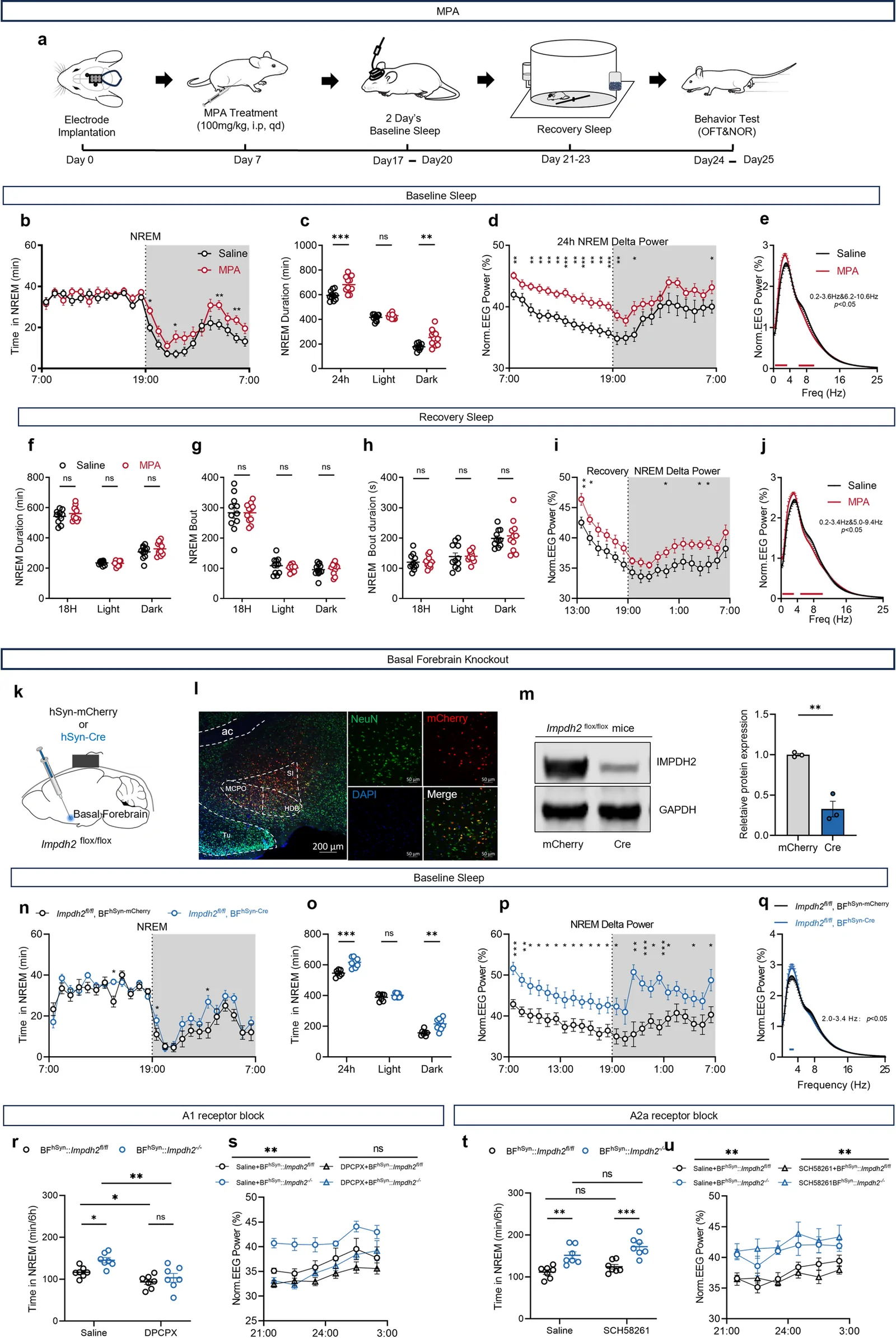
Basal forebrain IMPDH2 controls sleep-wake homeostasis via local adenosine mechanisms. **a** Schematic of the experimental design (*n* = 11 per group). **b** Hourly time spent in NREM sleep. **c** Quantification of total NREM sleep duration. **d** Hourly relative EEG delta power during NREM sleep. **e** Power spectral density of NREM sleep. **f–h** Quantification of NREM sleep parameters during recovery following sleep deprivation, including total NREM duration (**f**), NREM bout number (**g**), and single NREM bout duration (**h**). **i** Hourly relative NREM delta power during recovery sleep. **j** Relative power spectral distribution of NREM sleep during recovery sleep. **k** Experimental design of AAV-mediated Impdh2 deletion in BF neurons. Impdh2^fl/fl^ mice received bilateral BF injections of either AAV-hSyn-Cre (knockout, *n* = 8) or AAV-hSyn-mCherry (control, *n* = 7). **l** Representative images confirming viral expression in the BF. **m** Immunoblot confirming efficient deletion of IMPDH2 in BF neurons (*n* = 3 per group). **n** Hourly time spent in NREM sleep during baseline recording. **o** Quantification of total NREM sleep duration. **p** Hourly relative EEG delta power during NREM sleep. **q** Power spectral density of NREM sleep. **r** Quantification of NREM sleep duration after DPCPX injection through BF cannula in control and Impdh2 BF Neu-cKO mice (*n* = 7 per group). **s** Hourly relative EEG delta power during NREM sleep after DPCPX injection. **t** Quantification of NREM sleep duration after SCH58261 injection through BF cannula in control and Impdh2 BF Neu-cKO mice (*n* = 7 per group). u. Hourly relative EEG delta power during NREM sleep after SCH58261 injection. Data are presented as mean ± SEM

Our cellular analyses implicate IMPDH2 in BTBD9-mediated adenosine metabolism, to assess the functional relevance of this axis for sleep, we manipulated *IMPDH2 in vivo* and examined sleep-wake architecture. We first chronically treated wild-type mice with the IMPDH2 inhibitor MPA (50 mg/kg/day) *via* intraperitoneal injection for two weeks [42] (Fig. 4a), which significantly increased baseline NREM sleep duration, predominantly during the active dark phase (Fig. 4b, c; see Fig. S9 for complete baseline sleep architecture). The treatment concurrently enhanced SWA (Fig. 4d, e). Moreover, following sleep deprivation, MPA treated mice maintained higher SWA during recovery (Fig. 4f-j). Behavioral assessment revealed that MPA-treated mice exhibited reduced center transitions in the OFT (Fig. S10a), while the NOR performance remained intact (Fig. S10b), indicating preserved cognitive function under this treatment paradigm.

The sleep-promoting effects of systemic IMPDH2 inhibition prompted us to examine its localization relative to BTBD9 in the human brain. Analysis of brain expression datasets revealed that *IMPDH2* exhibits a largely complementary distribution to *BTBD9* across the brain, yet both proteins show prominent expression in the human basal forebrain (Fig. S11a). This regional convergence further suggests the BF as a potential site of BTBD9–IMPDH2 functional interaction. After confirming *Impdh2* expression in mouse BF neurons (Fig. S11b), we generated *Impdh2*^fl/fl^ mice for BF neuron-specific deletion (Neu*-*cKO) (Fig. 4k-m). Neuronal *Impdh2* knockout in BF significantly increased NREM duration and SWA (Fig. 4n-q), particularly at night (Fig. 4n, o), primarily through increased NREM bout frequency (Fig. S12a), without altering mean NREM bout duration (Fig. S12b). Consistently, wakefulness was reduced (Fig. S12c-f), while REM sleep remained unchanged (Fig. S12g-j). Regarding behavioral performance, *Impdh2* knockout in BF mice exhibited similar alterations in the OFT and comparable improvements in cognitive function to *Btbd9* cKO mice, suggesting functionally aligned effects of IMPDH2 and BTBD9 on behavioral outcomes, consistent with our hypothesis that IMPDH2 is primarily regulated at the level of enzymatic activity rather than protein abundance. During recovery sleep, these *Impdh2* BF Neu*-*cKO mice showed further increased NREM sleep duration and SWA (Fig. S13a, b, f, g). Moreover, the sleep-promoting phenotype of *Impdh2* knockouts were completely abolished by A1R antagonist DPCPX, but unaffected by A2aR antagonist SCH58261, confirming the adenosine-A1R mediation (Fig. 4r-u). Given that IMPDH2 also controls guanine nucleotide synthesis and guanine metabolites have been implicated in neural signaling [43], we briefly examined whether GTP-related mechanisms might contribute to the observed sleep phenotype. However, neither systemic GTP administration nor local BF GTP injection altered sleep architecture (Fig. S14 and Fig. S15), effectively excluding a direct GTP-mediated mechanism and reinforcing adenosine as the principal downstream mediator of IMPDH2 in sleep regulation.

Considering the region-specific effects of adenosine on sleep-wake regulation across the brain, we next examined whether IMPDH2 similarly modulates adenosine-dependent sleep circuits in other regions: the ventrolateral preoptic area (VLPO) and the parabrachial zone (PZ), with VLPO function through both A1R and A2aR [44], and PZ adenosine promoting wakefulness [45]. Following confirming *Impdh2* expression in neurons of both regions (Fig. S16), we achieved PZ and VLPO neuron-specific knockout of *Impdh2*. PZ *Impdh2* Neu-cKO significantly reduced baseline NREM sleep and SWA (Fig. S17). After sleep deprivation, these mice exhibited greater NREM suppression and lower SWA throughout the day (Fig. S18), consistent with adenosine’s wake-promoting role in the PZ [45]. While in the VLPO, *Impdh2* Neu-cKO slightly increased REM sleep without affecting baseline NREM sleep (Fig. S19). However, post-sleep deprivation, VLPO *Impdh2* Neu-cKO reduced nighttime recovery sleep and extended mean wake bout duration (Fig. S20b, e), indicating a wake-promoting effect. Together, these findings identify *IMPDH2* as a physiologically relevant downstream effector that modulates sleep-wake architecture in vivo in a manner consistent with adenosine signaling rather than guanine nucleotide-mediated mechanisms, supporting a role for IMPDH2 in mediating, at least in part, the sleep-regulatory effects of *BTBD9* in the BF.

### BTBD9 and IMPDH2 regulate adenosine signaling and purine metabolism in the basal forebrain

**Fig. 5:**
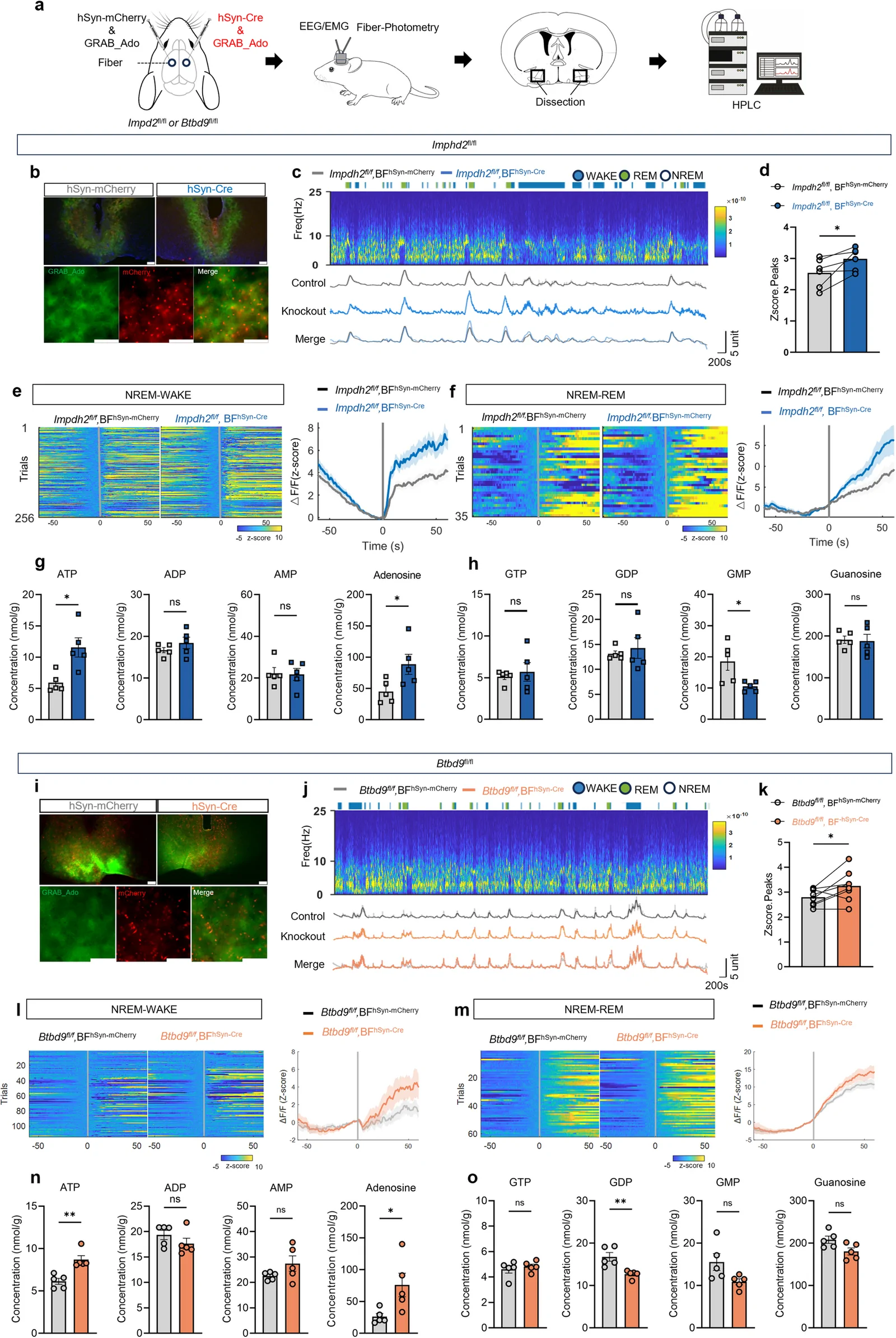
BTBD9 and IMPDH2 regulate adenosine signaling and purine metabolism in the basal forebrain. **a** Experimental design for in vivo adenosine monitoring. **b** Representative images validating AAV injection and fiber implantation in the BF. **c** Representative GRAB_Ado fluorescence traces comparing adenosine signaling in Impdh2^fl/fl^ mice injected in the BF with AAV-hSyn-Cre or AAV-hSyn-mCherry. **d** Quantification of adenosine signal peaks (*n* = 6 pairs). **e** Averaged GRAB_Ado responses aligned to NREM-wake transitions in control and Impdh2 BF Neu-cKO mice. **f** Averaged GRAB_Ado responses aligned to NREM-REM transitions in control and Impdh2 BF Neu-cKO mice. **g**,** h** Concentrations of purine-derived metabolites in BF tissues, including ATP, ADP, AMP, and adenosine (**g**) and GTP, GDP, GMP, and guanosine (**h**), measured from BF Impdh2 Neu-cKO and control sides (*n* = 5 per group). **i** Representative images validating AAV injection and fiber implantation in the BF of Btbd9^fl/fl^ mice. **j** Representative GRAB_Ado fluorescence traces comparing adenosine signaling in Btbd9^fl/fl^ mice injected in the BF with AAV-hSyn-Cre or AAV-hSyn-mCherry. **k** Quantification of adenosine signal peaks (n  = 9 pairs). **l.** Averaged GRAB_Ado responses aligned to NREM–wake transitions in control and Btbd9 BF Neu-cKO mice. **m** Averaged GRAB_Ado responses aligned to NREM–REM transitions in control and Btbd9 BF Neu-cKO mice. **n**,** o** Concentrations of purine-derived metabolites in BF tissue, including ATP, ADP, AMP, and adenosine (**n**) and GTP, GDP, GMP, and guanosine (**o**), measured from BF neuron–specific Btbd9 knockout and control sides (*n* = 5 per group). Data are presented as mean ± SEM

To examine the physiological impact of the BTBD9-IMPDH2 axis on adenosine dynamics in the BF, we performed fiber photometry imaging of GRAB_Ado during sleep-wake transitions in mice. We first performed unilateral injection of control virus and AAV-h*Syn*-Cre into the left and right BF of *Impdh2*^fl/fl^ mice, enabling within-animal comparison of control and knockout conditions (Fig. 5a and S21). Fiber photometry recordings (Fig. 5b) revealed that extracellular BF adenosine levels significantly increased during transitions from NREM to wake and REM states (Fig. 5c), consistent with the known adenosine dynamics [27]. Notably, *Impdh2* knockout elevated the signal peaks during these sleep state transitions (Fig. 5d-f).

Using the same unilateral genetic knockout approach, we measured the calcium dynamics via fiber photometry and found that *Impdh2* knockout did not significantly alter calcium dynamics during sleep state transitions (Fig. S22), ruling out neuronal excitability as the cause of elevated adenosine levels and altered sleep architectures in *Impdh2* knockouts. Targeted metabolomic analysis of the BF tissues further revealed that *Impdh2* deficiency significantly elevated adenosine levels (Fig. 5g) and concomitantly reduced GMP levels (Fig. 5h), consistent with IMPDH2’s role in guanine nucleotide biosynthesis. Taken together, these findin\gs establish that IMPDH2 regulates sleep through purine metabolic reprogramming rather than neuronal activity changes.

We next monitored extracellular adenosine dynamics in BF neuron–specific *Btbd9* knockout mice to provide in vivo physiological support for the proposed BTBD9–IMPDH2 pathway (Fig. 5i). Similar to *Impdh2* cKO, *Btbd9* deletion leads to an increased extracellular BF adenosine release during state transitions (Fig. 5j–m), accompanied by elevated tissue adenosine levels (Fig. 5n) and reduced guanosine levels, as revealed by the metabolic profiling assay (Fig. 5o). This metabolic reprograming was consistent with the increased NREM sleep observed in *Btbd9* BF Neu-cKO mice. Together with the IMPDH2 manipulations, these results provide in vivo physiological support for the proposed BTBD9–IMPDH2 pathway in regulating adenosine signaling and sleep–wake architecture in the BF.

### BTBD9-dependent regulation of IMPDH2 engages adenosine signaling to control sleep in the basal forebrain

**Fig. 6:**
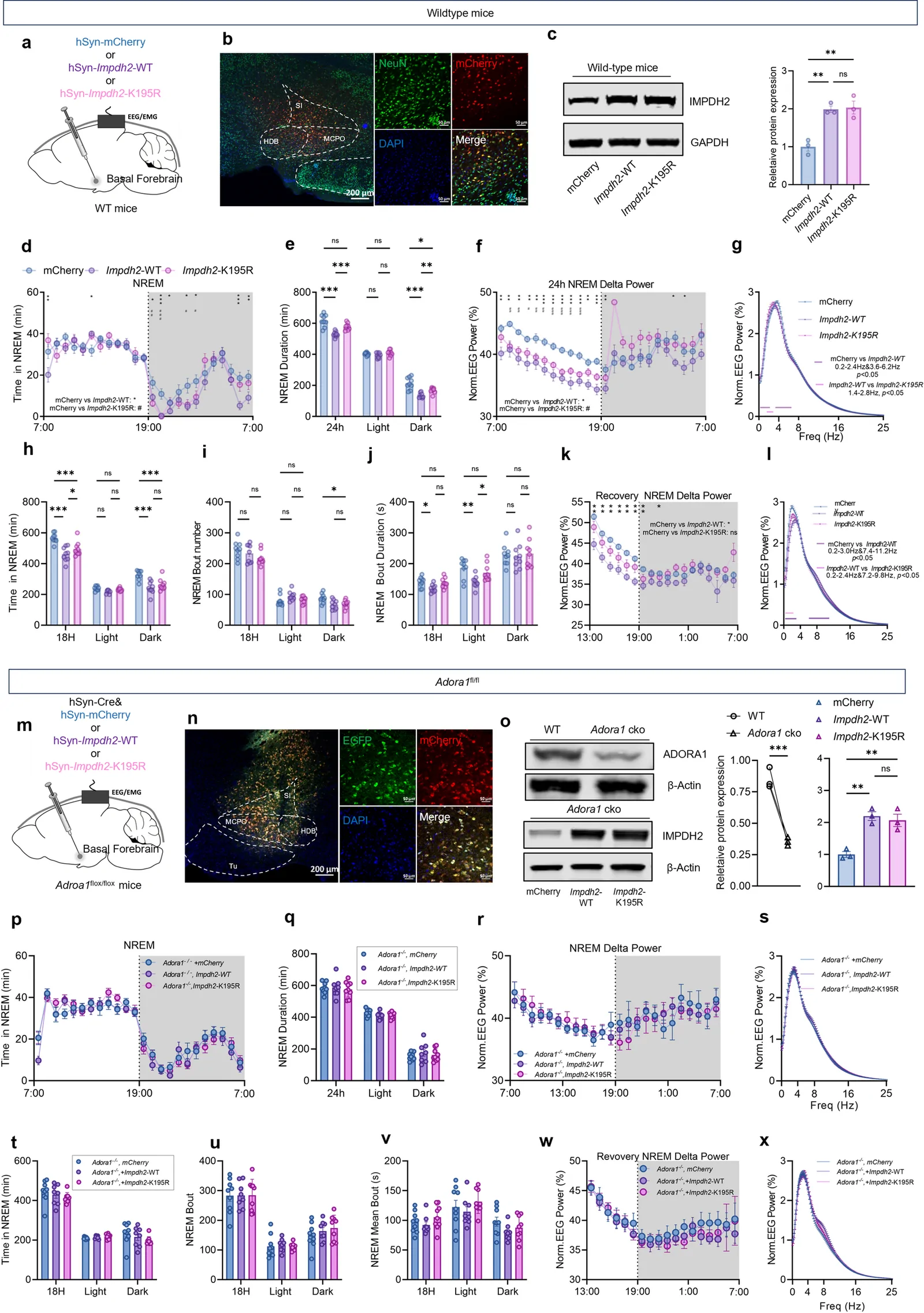
BTBD9-dependent regulation of IMPDH2 engages adenosine signaling to control sleep in the basal forebrain. **a** Experimental schematic illustrating AAV-mediated overexpression of mCherry, Impdh2-WT, or Impdh2-K195R in BF neurons of wild-type mice (*n* = 9 per group). **b** Representative fluorescence images showing NeuN labeling and hSyn-driven Impdh2-WT expression in the BF. **c** Western blot analysis confirming Impdh2 expression in the BF of control, Impdh2-WT, and Impdh2-K195R groups (*n* = 3 per group). **d–g** Baseline sleep architecture following Impdh2-WT or Impdh2-K195R overexpression, including hourly NREM sleep duration (**d**), total NREM sleep (**e**), relative NREM delta power (**f**), and NREM power spectral density (**g**). **h–l** Recovery sleep following sleep deprivation, including total NREM duration (**h**), NREM bout number (**i**), mean NREM bout duration (**j**), hourly relative NREM delta power during recovery (**k**), and NREM power spectral density (**l**). **m–o** Schematic and validation of basal forebrain specific Adora1 Neu-cKO mice (*n* = 9 for mCherry and Impdh2-K195R group, *n* = 8 for Impdh2-WT). **p–s** Baseline sleep architecture in Adora1 Neu-cKO mice following mCherry, Impdh2-WT or Impdh2-K195R overexpression, including NREM sleep duration, NREM sleep duration and power spectral density. **t–x** Recovery sleep following sleep deprivation in Adora1 Neu-cKO mice, including NREM duration, NREM bout number, NREM bout duration and delta power dynamics. Data are presented as mean ± SEM

To further identify whether BTBD9-dependent ubiquitination of IMPDH2 contributes to sleep control in vivo, we compared the effects of BF overexpression of wild-type *Impdh2* (*Impdh2*-WT) and its K195R mutant (*Impdh2*-K195R) (Fig. 6a–c). Although both constructs reduced NREM sleep in the dark phase (Fig. 6d, e) and NREM delta power (Fig. 6f, g) under baseline conditions (see also WAKE and REM sleep in Fig. S23c–j), the K195R mutation consistently attenuated the alterations in sleep architecture, including changes in NREM bout number and mean bout duration (Fig. S23a, b). Notably, *Impdh2-*WT expression significantly suppressed homeostatic recovery following sleep deprivation, as reflected by reduced NREM sleep, decreased NREM mean bout duration, and attenuated SWA during the 6 h recovery period, whereas these effects were largely rescued in mice expressing the K195R mutant (Fig. 6h–l). In behavioral assays, *Impdh2*-WT overexpression increased locomotor activity in the OFT, with more entries into the central zone, whereas the *Impdh2-*K195R group showed no significant differences compared with controls (Fig. S23k). Neither construct affected cognitive performance in the NOR test (Fig. S23l). Together, these results indicate that BTBD9-regulated ubiquitination of IMPDH2 serves as a critical regulatory molecular switch that determines the potency of IMPDH2 in modulating sleep-wake behavior.

To further determine whether the sleep-regulatory effects of IMPDH2 ubiquitination require local adenosine-A1R signaling, we co-injected AAV-h*Syn*-Cre and AAVs expressing either *Impdh2*-WT or *Impdh2*-K195R into the BF of *Adora1*^fl/fl^ mice (Fig. 6m-n). This strategy allowed us to simultaneously induce the Neu-cKO of *Adora1* and the neural-specific overexpression of IMPDH2 variants within the BF circuit. (Fig. 6o). Notably, the sleep-modulating capacity of IMPDH2 was completely abolished in the absence of A1R. Specifically, *Impdh2*-WT failed to further alter NREM sleep duration, and more importantly, the functional disparity previously observed between the WT and the K195R mutant was entirely eliminated (Fig. 6p–s; Fig. S24). Both constructs yielded indistinguishable NREM sleep patterns during both baseline and post-sleep deprivation recovery (Fig. 6t–x), as well as behavior performance (Fig. S24k, l). These findings demonstrate that the BTBD9-IMPDH2 axis orchestrates sleep homeostasis specifically through the adenosine-A1R signaling pathway within BF circuits.

### Dissociation of the IMPDH2-BTBD9 complex promotes sleep

**Fig. 7:**
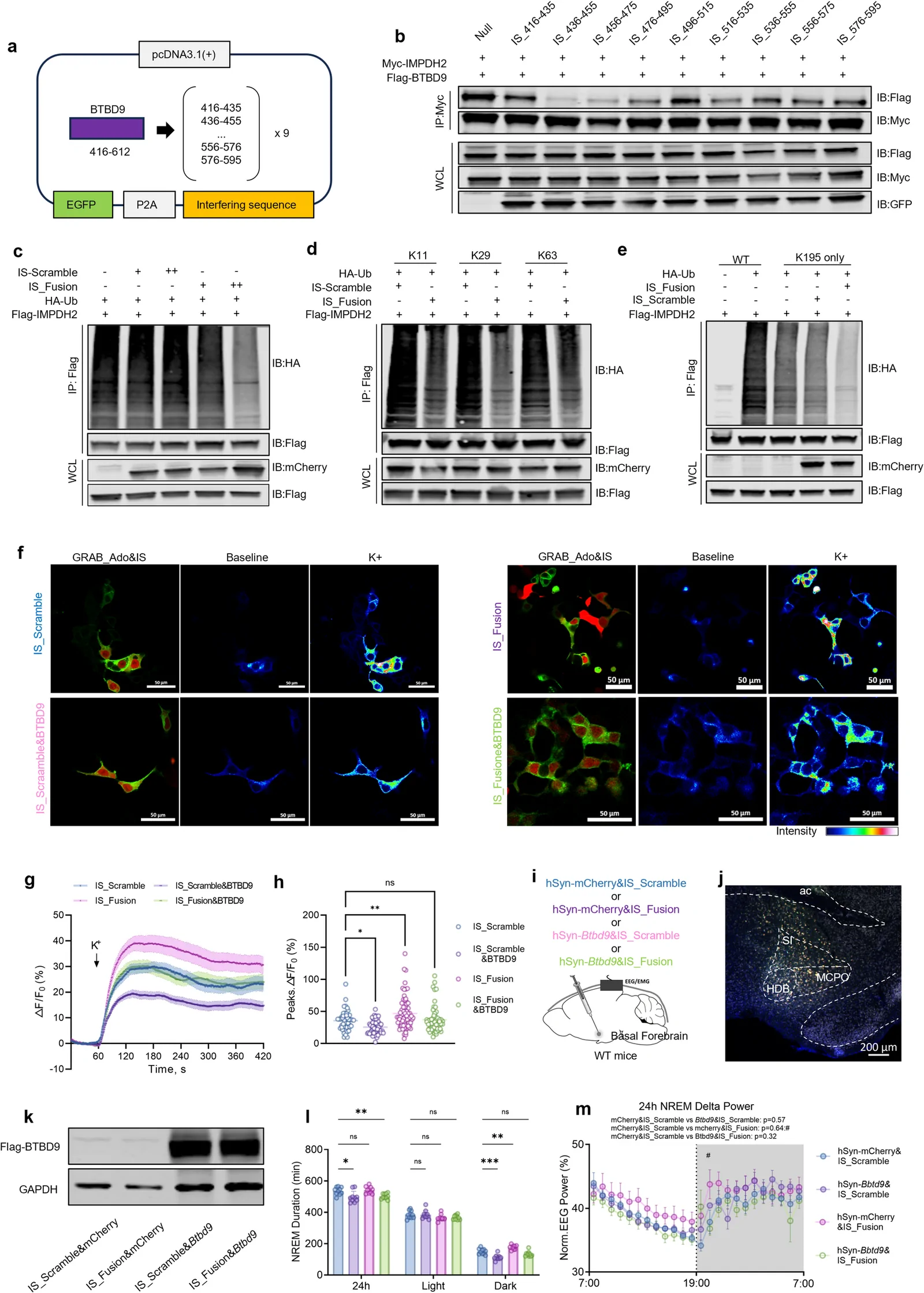
Dissociation of the IMPDH2-BTBD9 complex promotes sleep. **a** Schematic illustration of the design of interfering sequence constructs targeting the BTBD9–IMPDH2 interaction. **b** Co-IP assays of HEK293T cells expressing Flag-BTBD9 and Myc-IMPDH2 with different interfering sequence constructs, assessed by IP and immunoblotting with anti-Flag, anti-Myc, and anti-GFP antibodies. **c** Ubiquitination assays in HEK293T cells expressing HA-Ub and Flag-IMPDH2 with IS-Scramble or IS-Fusion constructs, analyzed by IP and immunoblotting with anti-Flag, anti-HA, and anti-mCherry antibodies. **d** Ubiquitination assays examining different ubiquitin linkages (K11-Ub, K29-Ub, and K63-Ub) in HEK293T cells expressing Flag-IMPDH2 and IS-Fusion constructs. **e** Ubiquitination assays of HEK293T cells expressing HA-Ub, Flag-IMPDH2 or the Flag-IMPDH2-K195 mutant, and IS-Fusion constructs, analyzed by IP and Western blotting. **f** Representative GRAB_Ado fluorescence images showing adenosine signals in cells expressing IS-Scramble or IS-Fusion with or without BTBD9 overexpression following KCl stimulation. **g** Time-course traces of GRAB_Ado signals in response to KCl stimulation across the four experimental groups. **h** Quantification of peak adenosine responses following KCl stimulation (IS-Scramble *n* = 48 ROIs, IS-Fusion *n* = 70 ROIs, IS-Scramble + BTBD9 *n* = 56 ROIs, IS-Fusion + BTBD9 *n* = 55 ROIs). **i** Schematic illustration of AAV-mediated co-expression of interfering sequences and Btbd9 in BF neurons of mice (*n* = 10 per group). **j** Representative fluorescence images showing GFP, mCherry, and NeuN labeling in the basal forebrain verifying AAV injection. **k** Western-blotting confirming Btbd9 overexpression in the different experimental groups. **l** Quantification of total NREM sleep duration across experimental groups. **m** Hourly relative NREM delta power during sleep recording. Data are presented as mean ± SEM

To establish that the physical association between BTBD9 and IMPDH2 is the prerequisite for BTBD9-mediated sleep regulation, we employed a peptide-based interference strategy to pharmacologically dissociate this complex in vivo. Based on our previous mapping, which localized the IMPDH2-binding region to amino acids 416–612 of BTBD9 (Fig. 2k), we rationally designed nine interfering sequences, each encoding a 20-amino-acid peptide fragment spanning this region (designated as interfering sequences, prefixed “IS-”) (Fig. 7a). Co-IP experiments revealed that two adjacent peptides (IS_436–455 and IS_456–475) disrupted the BTBD9-IMPDH2 interaction (Fig. 7b), without increasing endogenous IMPDH2 expression (Fig. S25a). Since these two consecutive peptides were contiguous, we generated a fusion construct encompassing this 40-amino-acid region (IS_Fusion; residues 436–475 of BTBD9) to maximize the interference effect. Expression of IS_Fusion reduced IMPDH2 ubiquitination (Fig. 7c), including the previously identified K11-, K29-, and K63-linked ubiquitin chains (Fig. 7d), and critically diminished ubiquitination at the IMPDH2-K195 site (Fig. 7e), the principal BTBD9-regulated modification. Despite reduced ubiquitination, endogenous IMPDH2 protein levels were modestly decreased (Fig. S25b), reinforcing our earlier finding that BTBD9-dependent modification primarily modulates IMPDH2 enzymatic potency rather than its proteasomal stability.

Consistent with the role of the BTBD9-IMPDH2 in gating adenosine, disrupting their physical association significantly altered adenosine dynamics. Using the GRAB_Ado [34] sensor in HEK293T cells, we observed that IS_Fusion not only effectively restored the adenosine suppression induced by *BTBD9* overexpression but also independently increased extracellular adenosine levels (Fig. 7f-h). These findings confirm that the physical association between BTBD9 and IMPDH2 is essential for maintaining cellular adenosine homeostasis.

To determine whether dissociation of the IMPDH2-BTBD9 complex regulates sleep in vivo, we overexpressed *Btbd9* alone or together with IS_Fusion in the BF of mice (Fig. 7i–k, Fig. S26). IS_Fusion was delivered via an adeno-associated viral vector (rAAV-hSyn-EGFP-P2A-IS_Fusion) to achieve a relative long-term expression, and successful expression in basal forebrain neurons was verified by EGFP fluorescence, which served as a surrogate marker for IS_Fusion expression(Fig. S26). *Btbd9* overexpression significantly reduced NREM sleep over 24 h, particularly during the dark phase (Fig. 7l). Co-expression of IS_Fusion abolished this nocturnal NREM suppression (Fig. 7l) without affecting SWA (Fig. 7m). Notably, expression of IS_Fusion alone increased NREM sleep during the dark period (Fig. 7i–k, Fig. S27), suggesting that disruption of the BTBD9–IMPDH2 interaction may bias sleep–wake balance toward increased sleep propensity under physiological conditions. During sleep deprivation, IS_Fusion alone did not significantly affect baseline NREM rebound but partially rescued the impaired recovery sleep induced by *Btbd9* overexpression (Fig. S28a–d). In behavioral assays, IS_Fusion did not significantly alter locomotor activity in the OFT (Fig. S28f), but alleviated *Btbd9* overexpression-induced cognitive deficits in the NOR task (Fig. S28e).

Collectively, our results establish that the BTBD9-IMPDH2-adenosine axis constitutes a fundamental mechanistic pathway for sleep homeostasis. By disrupting this coupling, we effectively uncoupled BTBD9 from its downstream metabolic effectors, thereby defining the BTBD9-mediated ubiquitination of IMPDH2 as a primary driver of arousal-promoting signaling.

## Discussion

**Fig. 8:**
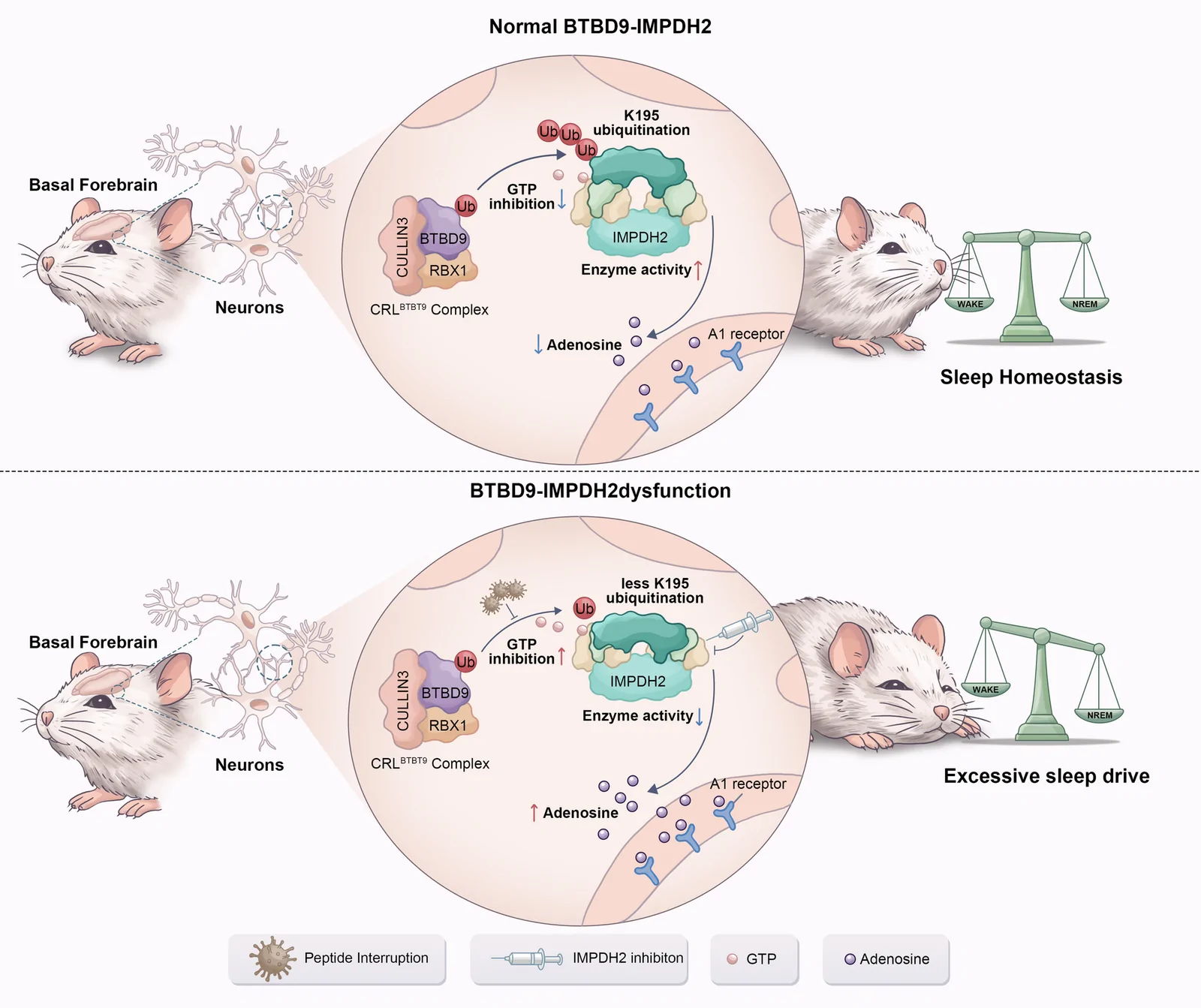
Model of the BTBD9–IMPDH2–adenosine pathway regulating sleep homeostasis. In the mice basal forebrain neurons, BTBD9 promotes K195 ubiquitination of IMPDH2 via the CRL3 complex, thereby reducing GTP binding to IMPDH2 and attenuating its feedback inhibition. This enhances IMPDH2 activity and purine flux, leading to increased adenosine production, which acts through adenosine A1 receptors to regulate sleep homeostasis

Although *BTBD9* has been repeatedly implicated in human GWAS of sleep disorders [8, 10, 13] and is widely considered an evolutionarily conserved sleep regulator, it is still unclear how genetic variation in *BTBD9* contributes to sleep phenotypes in humans, and the molecular mechanism by which BTBD9 protein regulates sleep is also poorly understood. In this study, by combining human genetic analyses with circuit-specific manipulations in the mouse BF, we first identify *BTBD9* as an arousal promoting factor that regulates sleep homeostasis. Through cross-species ubiquitinomics, structural analyses, and in vivo functional experiments, we further show that BTBD9 controls sleep by regulating the enzymatic activity of IMPDH2 rather than its abundance. This interaction modulates purine metabolism in the BF and ultimately influences NREM sleep through adenosine A1 receptor signaling. Together, these findings provide a mechanistic link between human *BTBD9* genetic variation, purine metabolism, and sleep circuit function (Fig. 8).

Our study unveils a non-canonical mode of ubiquitin-dependent sleep regulation that differs from established paradigms. While sleep research has extensively focused on transcriptional and neuromodulatory mechanisms [46–50], the role of post-translational protein homeostasis, specifically ubiquitination, remains comparatively under-explored. Previously, CRL3-based E3 ligases were primarily known to regulate sleep and circadian processes through canonical ubiquitin-proteasome-mediated degradation, as exemplified by the degradation of substrates such as insomniac [21] and TIMELESS [20]. In contrast, our findings establish IMPDH2 as a neuronal substrate whose functional output is primarily orchestrated by CRL3^BTBD9^-mediated ubiquitination at the K195 site, rather than through changes in protein abundance. Although BTBD9-dependent ubiquitination occurs within a broader multi-site landscape, where different lysine residues may differentially contribute to IMPDH2 stability and function, our functional assays using IMPDH2 lysine mutants revealed that the K195 mutant altered adenosine levels differently from other mutants and wild-type IMPDH2 when expressed at comparable protein levels (Fig. 3r and s). These K195-mediated changes in enzymatic activity are consistent with the sleep-promoting phenotype observed upon *Btbd9* deletion in the mouse basal forebrain. These data support a site-specific role for K195 in modulating IMPDH2 enzymatic activity, likely through allosteric or conformational mechanisms. Specifically, we show that BTBD9-dependent ubiquitination at K195 serves as a regulatory switch that relieves the allosteric feedback inhibition of IMPDH2 by GTP [51]. This is accompanied by shifts in filament assembly, a structural hallmark of IMPDH2 enzymatic activation [23], indicating that BTBD9 primarily regulates IMPDH2 at the level of enzymatic function. This non-degradative mode of ubiquitin regulation provides a mechanism by which BTBD9 can tune neuronal purine metabolic flux and thereby influence downstream sleep-relevant metabolites. While combining proteomic and ubiquitinomic strategy successfully prioritized IMPDH2 as a high-confidence BTBD9 substrate, given that BTBD9–IMPDH2 primarily acts through non-degradative ubiquitination, additional substrates regulated without changes in protein levels may not be fully captured. Such neurodegeneration relevant targets may warrant further investigation in future studies.

*BTBD9* has emerged as a pleiotropic risk factor across diverse sleep disorders, yet a unifying mechanism has remained elusive. Here, we found that BF-specific Btbd9 deletion increased NREM sleep preferentially during the dark phase. This temporal specificity may reflect the interaction between homeostatic sleep pressure and circadian regulation of sleep–wake, rather than a simple change in sleep amount. Increased sleep pressure after Btbd9 deletion may be less sensitive translated to sleep amount change due to already abundant baseline sleep. Consistently, elevated NREM delta power during the light phase and early recovery after sleep deprivation supports enhanced sleep pressure even when total sleep duration was not markedly changed. However, the reduced wake bout duration during the dark phase also raises the possibility that impaired active-phase wake maintenance contributes to the increased NREM sleep. Further studies will be required to distinguish the relative contributions of increased sleep propensity, altered circadian gating, and impaired wake stability to this phase-specific phenotype. Together, our discovery that the BTBD9–IMPDH2 axis in BF dictates sleep homeostasis via adenosine metabolism provides a direct mechanistic link to the pathogenesis of insomnia and the abnormal arousals characteristic of OSA [8, 10, 13].

While we did not directly assess RLS, the IMPDH2-dependent metabolic shunt offers a compelling biochemical framework for its pathology. Given that IMPDH2-derived GTP production is essential for dopaminergic metabolism [52] and has been linked to iron-related pathways in RLS [53], the BTBD9–IMPDH2 axis may exert pleiotropic effects by modulating distinct metabolic outputs across brain regions in a circuit-dependent manner. By the way, this regional metabolic specificity, potentially arising from substrate- and brain region-dependent effects of BTBD9, may help reconcile the differences between global *Btbd9* knockout and BF-specific conditional knockout models. While global deletion of *Btbd9* is associated with both sleep disturbances and hyperactivity, BF-specific manipulation primarily promote sleep. Together, these findings support a model in which BTBD9 does not operate through a uniform brain-wide mechanism, but instead exerts region- and circuit-dependent regulation of sleep–wake behavior, with the basal forebrain representing one key node within a distributed regulatory network. Furthermore, the established role of adenosine in both sleep homeostasis and RLS pathogenesis [26, 33, 34, 44, 54, 55] further underscores the IMPDH2-gated purine pathway as a potential unifying mechanism underlying the diverse sleep disorders associated with BTBD9. Importantly, emerging evidence suggests that adenosine signaling in RLS is receptor subtype- and circuit-dependent rather than uniformly hypoadenosinergic. For example, A1 receptor signaling is primarily implicated in presynaptic modulation of cortico-striatal glutamatergic transmission, whereas A2A receptor upregulation in striatal circuits has been linked to impaired sensorimotor integration and dopaminergic imbalance [56, 57]. This receptor-specific organization provides a potential framework to reconcile the hypoadenosinergic hypothesis with region-specific adenosine alterations. Ultimately, while this model positions the BTBD9-IMPDH2 interaction as a metabolic switch, further studies are required to validate these regional-specific effector mechanisms.

Our findings suggest that the BTBD9–IMPDH2–adenosine pathway represents a conserved molecular mechanism operating across distinct neural circuits that regulate sleep. Rather than reflecting an invariant function, the BTBD9-IMPDH2 axis operates through a biochemically conserved mechanism, modulating adenosine metabolism, that yields distinct physiological outcomes depending on the regional role of adenosine itself. This principle is illustrated by our exploratory *Impdh2* manipulations outside the BF. In the parafacial zone (PZ), where GABAergic neuronal populations have been classically implicated in NREM sleep initiation and maintenance [58, 59], adenosine recently has been reported to exert a wake-promoting effects via A1 receptors that differ from its canonical sleep-promoting role [45]. In line with these findings, we found that *Impdh2* deletion in the PZ reduced NREM sleep. Together with our IMPDH2-dependent metabolic data, these observations further support a role for IMPDH2 in regulating sleep through adenosine signaling, although direct receptor-level manipulations were not performed in the present study. Conversely, in the ventrolateral preoptic area (VLPO), a canonical sleep-promoting nucleus where adenosine enhances sleep pressure [60, 61], *Impdh2* deletion did not alter baseline NREM sleep but impaired the homeostatic sleep response to deprivation. These results support that the effect of BTBD9-IMPDH2 on sleep might gated by the region-specific functional role of adenosine. However, owning to the BTBD9 manipulations and adenosine A1/A2 receptor blockade were not performed in these regions, the full BTBD9–IMPDH2–adenosine signaling cascade remains to be directly tested. In addition, while our study identifies the BF as a functionally validated site for *BTBD9*-dependent sleep regulation, it does not imply regional exclusivity. Future work using systematic region-by-region perturbations will be required to fully map the distributed neural circuits involved such as basal ganglia. The use of hSyn-driven Cre also introduces a limitation, as it results in *Btbd9* deletion across multiple neuronal subtypes within the BF. Given the cellular heterogeneity of the BF contributes differently to the adenosine releasing and sleep-wake regulation, future cell-type-specific manipulations will be required to furtherly identify the specific neuronal populations underlying the observed phenotype.

Notably, beyond adenosine, IMPDH2-mediated alterations in purine metabolism are expected to affect broader nucleotide pools, including ATP/ADP, AMP, GTP, GMP, and guanosine. In particular, while ATP and its breakdown product AMP are closely linked to sleep pressure by adenosine accumulation, ATP itself may play a role in sleep regulation independently via P2 receptors [62]. In addition, guanine nucleotide metabolism, including GTP, GMP, and guanosine, which have been implicated in neuronal excitability and neurodegenerative processes [63, 64] also have the potential to regulate sleep despite little evidence exist. Notably, GTP-derived pathways have been associated with dopaminergic metabolism [52], suggesting that IMPDH2-dependent regulation of guanine nucleotides may influence arousal-related circuits.

Beyond its mechanistic insights, this study establishes a framework that links human *BTBD9* genetic associations to the fundamental mechanisms of sleep regulation. By characterizing the BTBD9-IMPDH2-adenosine axis, we bridge GWAS-identified susceptibility signals with a validated biochemical pathway, revealing a circuit-dependent mode of action within the basal forebrain. Although this study focuses on fundamental mechanisms, identifying this conserved metabolic-circuit interaction provides a critical foundation for future translational studies of *BTBD9*-linked pathologies. Ultimately, this work significantly advances our understanding of *BTBD9* biology and contributes to the broader molecular and genetic landscape of sleep regulation.

## Supplementary Information

Supplementary Material 1. Supplementary Figures.

Supplementary Material 2. Table S1.

Supplementary Material 3. Table S2.

Supplementary Material 4. Table S3.

Supplementary Material 5. Table S4.

Supplementary Material 6. Table S5.

Supplementary Material 7. Table S6

Supplementary Material 8. Table S7.

## Data Availability

The proteomics datasets generated during the current study are available in the MassIVE repository (Accession IDs: PXD073875 and PXD073876). All other data supporting the findings of this study are included in the manuscript or its supplementary materials.

## Abbreviations

A1R: Adenosine A1 receptor
A2aR: Adenosine A2a receptor
AAV: Adeno-associated virus
ACTB: Actin beta
ADP: Adenosine diphosphate
AMP: Adenosine monophosphate
AP: Anteroposterior
As-cKO: Astrocyte-specific conditional knockout
ATP: Adenosine triphosphate
BF: Basal forebrain
BTB: Broad-complex, tramtrack and bric-à-brac
BTBD9: BTB domain containing 9
cKO: Conditional knockout
CHX: Cycloheximide
Co-IP: Co-immunoprecipitation
CRL3: Cullin-RING E3 ubiquitin ligase 3
CUL3: Cullin 3
DPCPX: 8-cyclopentyl-1,3-dipropylxanthine
DV: Dorsoventral
EEG: Electroencephalography
EMG: Electromyography
eQTL: Expression quantitative trait locus
FFT: Fast Fourier transform
GAPDH: Glyceraldehyde-3-phosphate dehydrogenase
GDP: Guanosine diphosphate
GFP: Green fluorescent protein
GMP: Guanosine monophosphate
GRAB_Ado: G-protein-coupled receptor activation-based adenosine sensor
GTEx: Genotype-Tissue Expression
GTP: Guanosine triphosphate
GWAS: Genome-wide association study
ICC: Immunocytochemistry
IHC: Immunohistochemistry
IMP: Inosine monophosphate
IMPDH2: Inosine monophosphate dehydrogenase 2
IP: Immunoprecipitation
IS: Interfering sequence
KO: Knockout
LSD: Least significant difference
MPA: Mycophenolic acid
mRNA: Messenger RNA
NAD⁺: Nicotinamide adenine dinucleotide
NADH: Reduced nicotinamide adenine dinucleotide
Neu-cKO: Neuron-specific conditional knockout
NOR: Novel object recognition
NREM: Non-rapid eye movement
OFT: Open field test
OSA: Obstructive sleep apnea
PDB: Protein Data Bank
PFA: Paraformaldehyde
PME: Particle mesh Ewald
PSD: Power spectral density
PZ: Parafacial zone
RBX1: Ring-box 1
REM: Rapid eye movement
RLS: Restless legs syndrome
SCH58261: 7-(2-phenylethyl)-5-amino-2-(2-furyl)-pyrazolo[4,3-e]-1,2,4-triazolo[1,5-c]pyrimidine
siRNA: Small interfering RNA
SNP: Single nucleotide polymorphism
SWA: Slow-wave activity
UKB: UK Biobank
Ub: Ubiquitin
Vmax: Maximum reaction velocity
VLPO: Ventrolateral preoptic area
XMP: Xanthosine monophosphate
