# Supplementary material for: BTBD9-driven IMPDH2 ubiquitination governs sleep homeostasis by gating adenosine metabolism in the basal forebrain
Source: Cell Commun Signal. 2026 Jul 30;24:580. doi: 10.1186/s12964-026-03117-2 (PMC13711913; doi:10.1186/s12964-026-03117-2)
Supplement: Supplementary file 1 [file 12964_2026_3117_MOESM1_ESM.docx]

**Supplementary file for**

**BTBD9-Mediated IMPDH2 Ubiquitination Governs Sleep Homeostasis by Gating Adenosine Metabolism in the Basal Forebrain**

Zhenfei Gao^1,2,#^, Niannian Li^1,2,#^, Jiawei Feng^1,2,#^, Jinhong Shen^1,2^, Haolin Yuan^1,2^, Kejia Wu^1,2^, Xu Xu^1,2^, Yuenan Liu^3^, Yiming Hu^1,2^, Shengming Wang^1,2^, Hui Wang^1,2,*^, Xiangshan Yuan^4,*^, Jian Guan^1,2,*^, Feng Liu^1,2,*^, Shankai Yin^1,2,*^

1. Department of Otolaryngology-Head and Neck Surgery & Center of Sleep Medicine, Otolaryngology Institute of Shanghai Jiao Tong University, Shanghai Jiao Tong University School of Medicine Affiliated Sixth People's Hospital, Shanghai 200233, China.
2. Center for Sleep Monitoring and Respiratory Therapy Technology, Institute of Medical Equipment and Technology, Shanghai Jiao Tong University School of Medicine, Shanghai 200233, China.
3. Department of Otolaryngology & Head and Neck Surgery, Ruijin Hospital, Shanghai Jiao Tong University, School of Medicine, No. 197 Ruijin 2nd Road, Shanghai 200025, China

4. Department of Anatomy and Histoembryology, School of Basic Medical Sciences, Fudan University, Shanghai 200032, China.

\# ZG, NL, and JF share the first author position.

* Address correspondence to: Dr Shankai Yin, [skyin@sjtu.edu.cn,](mailto:skyin@sjtu.edu.cn,) Dr. Feng Liu, [liufeng@sibs.ac.cn](mailto:liufeng@sibs.ac.cn); Dr. Jian Guan, guanjian0606@sina.com; Dr. Xiangshan Yuan, yuanxiangshan1999@163.com，and Dr. Hui Wang, wangh2005@alumin.sjtu.edu.cn

**Fig. S1 *BTBD9* brain eQTL analysis linking expression direction to human sleep phenotypes.**

**a.** Integration of insomnia GWAS signals with GTEx brain eQTL data for *BTBD9*-associated variants. The x-axis represents the normalized effect size (NES) of BTBD9 expression in brain tissues, and the y-axis shows the GWAS effect size (beta) for insomnia risk. The color scale indicates GWAS significance, and dot size represents the significance of the eQTL association. **b.** Association between brain eQTL effects on *BTBD9* expression and daytime sleepiness using UK Biobank data; dot size represents eQTL significance, and color indicates FDR-adjusted eQTL significance. **c.** Luciferase reporter assays testing the regulatory activity of the OSA-related abnormal arousal risk variant rs117733138 (G>A). Constructs containing the reference allele (Ref-G) or risk allele (Alt-A) were compared with a Basic vector control in SHSY-5Y, 293T, and N2a cells. Sample sizes were n = 5 (Basic), n = 4 (Ref-G), and n = 4 (Alt-A) in SHSY-5Y cells; n = 6 (Basic), n = 4 (Ref-G), and n = 4 (Alt-A) in 293T cells; and n = 6 per group in N2a cells.

**Fig. S2 *BTBD9* expression pattern in the human and mice brain**

**a**. Heatmaps showing *BTBD9* mRNA expression across human brain regions based on data from the Allen Human Brain Atlas (6 donors). Each row represents a probe targeting *BTBD9*, and the color scale indicates normalized expression levels (red, higher expression; blue, lower expression). The top horizontal axis denotes major brain regions. **b.** Representative fluorescence image showing *Btbd9* mRNA distribution in the mouse brain. **c.** Representative fluorescence image showing *Btbd9* mRNA colocalization with NeuN-positive neurons in the mouse basal forebrain. **d**. Representative fluorescence image showing *Btbd9* mRNA colocalization with S100β-positive cells in the mouse basal forebrain.

**Fig. S3 Basal forebrain Neu-cKO of *Btbd9* alters sleep architecture.**

**a–b.** Quantification of NREM sleep bout structure, including NREM bout number (**a**) and mean NREM bout duration. (**b**). **c.** Hourly time spent in wakefulness across the recording period. **d–f.** Quantification of wake parameters, including total wake duration (**d**), wake bout number (**e**), and mean wake bout duration (**f**). **g.** Hourly time spent in REM sleep across the recording period. **h–j.** Quantification of REM sleep parameters, including total REM duration (**h**), REM bout number (**i**), and mean REM bout duration (**j**). Sample size: **n = 10** mice per group for panels **a–j**. Data are presented as mean ± SEM.

**Fig. S4 Basal forebrain As-cKO of *Btbd9* does not alter baseline sleep.**

**a.** Schematic illustrating AAV-mediated delivery of GfaABC1D-mCherry into the BF to target astrocytes. **b.** Representative fluorescence images showing S100β immunolabeling and GfaABC1D-mCherry expression in the BF, confirming astrocyte targeting. **c.** Quantitative RT-PCR analysis of *Btbd9* knockout efficiency in BF astrocytes (**n = 3** mice per group). **d-g.** Wake parameters in astrocyte-specific *Btbd9* knockout and control mice, including hourly time spent in wakefulness (**d**), total wake duration (**e**), wake bout number (**f**), and mean wake bout duration (**g**). **h–k.** NREM sleep parameters, including hourly NREM sleep duration (**h**), total NREM duration (**i**), NREM bout number (**j**), and mean NREM bout duration (**k**). **l-o.** REM sleep parameters, including hourly REM sleep duration (**l**), total REM duration (**m**), REM bout number (**n**), and mean REM bout duration (**o**). **p-q.** NREM EEG spectral features, including hourly NREM delta power (**p**) and NREM power spectral density (**q**). Sample size: control group, **n = 5** mice; astrocyte-specific *Btbd9* knockout group, **n = 6** mice. Data are presented as mean ± SEM.

**Fig. S5 Effect of basal forebrain As-cKO of *Btbd9* on recovery sleep.**

**a.** Hourly time spent in NREM sleep during the recovery period following sleep deprivation in control and astrocyte-specific *Btbd9* knockout mice. **B-d.** Quantification of NREM sleep parameters during recovery sleep, including total NREM duration (**b**), NREM bout number (**c**), and mean NREM bout duration (**d**). **e.** Mean wake bout duration across recovery sleep time stages. **f.** Hourly relative EEG delta power during NREM sleep in the recovery period. **g.** Power spectral density of NREM sleep during recovery sleep. Sample size: control group, **n = 5** mice; astrocyte-specific *Btbd9* knockout group, **n = 6** mice. Data are presented as mean ± SEM.

**Fig. S6 Proteomic and ubiquitinomic profiling of *Btbd9* knockout mice**

**a.** Volcano plot showing differential ubiquitination sites in the ubiquitinome comparing *Btbd9* KO and WT samples. Differentially regulated ubiquitination sites are highlighted as red based on fold change (FC>1.2) and statistical significance. (p<0.05). **b.** Heatmap of differentially regulated ubiquitination sites between WT and *Btbd9* KO samples. **c.** Volcano plot showing differentially expressed proteins in the proteome comparing *Btbd9* KO and WT samples. Differentially expressed proteins were filterd based on fold change (FC>1.2) and statistical significance (p<0.05). **d.** Heatmap of differentially expressed proteins between WT and *Btbd9* KO samples.

**Fig. S7 Molecular dynamics simulations of wild-type and K195-ubiquitinated IMPDH2.**

**a.** Visual inspection of the simulation snapshots revealed that the IMPDH2-K195-Ub complex retained an overall stable conformation throughout the 100-ns simulation. **b.** Root Mean Square Deviation (RMSD) of backbone atoms over a 100 ns simulation. The RMSD rapidly increases during the initial 10 ns, indicating structural adaptation, and then stabilizes around 1.3–1.7 nm, suggesting that the complex reaches equilibrium and maintains conformational stability throughout the simulation. **c.** Radius of Gyration (RG) analysis of the complex over time. The RG shows a slight decrease and then fluctuates around 2.9-3.0 nm, indicating consistent compactness of the protein structure and absence of major unfolding events, thereby supporting the overall structural reasonability of the IMPDH2-ub complex model.

**Fig. S8 Detailed molecular docking results.**

**a-c.** Docking conformation interaction analysis and docking conformation bonding analysis of IMPHD2-WT (a), IMPDH2-K195R(b), IMPDH2-K195-Ub(c).

**Fig. S9 Inhibition of IMPDH2 by MPA promotes NREM sleep.**

**a–b.** Quantification of NREM sleep bout structure, including NREM bout number (**a**) and mean NREM bout duration (**b**). **c.** Hourly time spent in wakefulness across the recording period. **d–f.** Quantification of wake parameters, including total wake duration (**d**), wake bout number (**e**), and mean wake bout duration (**f**). **g.** Hourly time spent in REM sleep across the recording period. **h–j.** Quantification of REM sleep parameters, including total REM duration (**h**), REM bout number (**i**), and mean REM bout duration (**j**). Sample size: **n = 11** mice per group for panels **a–j**. Data are presented as mean ± SEM.

**Fig. S10 Effect of MPA treatment on mice behavioral performance.**

**a.** Open field test assessing locomotor activity, including total distance traveled, distance in the center, time spent in the center, and transitions between center and peripheral areas in vehicle- and MPA-treated mice. **b.** Cognitive performance of vehicle- and MPA-treated mice assessed by NOR. Sample size: **n = 11** mice per group for panels. Data are presented as mean ± SEM.

**Fig. S11 IMPDH2 expression in neurons and astrocytes of the mouse basal forebrain.**

**a.** Heatmaps comparing *IMPDH2* mRNA expression across human brain regions with *BTBD9* based on data from the Allen Human Brain Atlas (6 donors). Each row represents a probe targeting IMPDH2, and the color scale indicates normalized expression levels (red, higher expression; blue, lower expression). The top horizontal axis denotes major brain regions. **b.** Representative fluorescence images showing IMPDH2 colocalization with NeuN-positive neurons in the mouse basal forebrain. Scale bar, 50 μm.

**Fig. S12 Effect of basal forebrain Neu-cKO of *Impdh2* on baseline sleep.**

**a.** Quantification of NREM bout number in control and basal forebrain neuron-specific *Impdh2* conditional knockout (cKO) mice. **b.** Mean NREM bout duration. **c.** Hourly time spent in wakefulness across the recording period. **d-f.** Quantification of wake parameters, including total wake duration (**d**), wake bout number (**e**), and mean wake bout duration (**f**). **g.** Hourly time spent in REM sleep across the recording period. **h-j.** Quantification of REM sleep parameters, including total REM duration (**h**), REM bout number (**i**), and mean REM bout duration (**j**). Sample size: **n = 8** mice in the *Impdh2* BF neuron–cKO group and **n = 7** mice in the control group for panels **a–j**. Data are presented as mean ± SEM.

**Fig. S13 Effect of basal forebrain Neu-cKO of *Impdh2* on recovery sleep and behavioral performance.**

**a.** Hourly time spent in NREM sleep during recovery following sleep deprivation. *Impdh2* BF Neu-cKO group, **n=8** mice; control group, **n=7** mice (same groups as in **b–g**). **b–d.** Quantification of NREM sleep parameters during recovery sleep, including total NREM duration (**b**), NREM bout number (**c**), and mean NREM bout duration (**d**). **e.** Mean wake bout duration across recovery sleep time stages. **f.** Hourly relative EEG delta power during recovery NREM sleep. **g.** Power spectral density of SWA during recovery NREM sleep. **h–i.** Quantification of OFT and NOR performance (**n=12** mice per group). Data are presented as mean ± SEM.

**Fig. S14 Effect of systemic GTP administration on sleep architecture.**

**a-d.** Wake parameters following intraperitoneal GTP injection, including hourly time spent in wakefulness (**a**), total wake duration over the 6-h recording period (**b**), wake bout number (**c**), and mean wake bout duration (**d**). **e-h.** NREM sleep parameters, including hourly NREM sleep duration (**e**), total NREM duration (**f**), NREM bout number (**g**), and mean NREM bout duration (**h**). **i–l.** REM sleep parameters, including hourly REM sleep duration (**i**), total REM duration (**j**), REM bout number (**k**), and mean REM bout duration (**l**). **m.** Hourly relative EEG delta power during NREM sleep following GTP administration. **n.** Power spectral density of NREM sleep. Saline and GTP were administered at 09:00 using a paired experimental design (**n=7** pairs). Data are presented as mean ± SEM.

**Fig. S15. BF local GTP administration did not affect sleep architecture and locomotor behavior.**

Saline (ACSF) or GTP (50 nM, 100 nl/side, 50 nl/min) was locally injected into the BF at 9:00 pm using a crossover design. Mice first received ACSF or GTP (***n* = 4 vs. 5**), and after a one-week washout period, treatments were switched (***n* = 5 vs. 4**). **a-c.** Hourly time spent in wake (**a**), NREM sleep (**b**), and REM sleep (**c**) during the 6 hours following local injection. **d–f.** Total time spent in wake (**d**), NREM (**e**), and REM (**f**) over the 6 hour post-injection period shown as paired comparisons within the same animals. **g.** Group-level comparison of total time spent in each vigilance state during the 6 hour period following ACSF or GTP injection. **h-k.** Open field test (OFT) performed after injection, including total distance traveled (**h**), distance traveled in the center (**i**), time spent in the center (**j**), and number of center–periphery transitions (**k**). Saline (ACSF) or GTP (50 nM, 100 nl/side, 50 nl/min) was locally injected into the basal forebrain (BF) at 21:00 using a crossover design. Mice first received ACSF or GTP (**n=4** vs **n=5**), and after a one-week washout period the treatments were switched (**n=5** vs **n=4**). Data are presented as mean ± SEM.

**Fig. S16 IMPDH2 expressed in the mouse neurons of VLPO AND PZ area**

**a,b.** Fluorescence image of NeuN, and IMPDH2 in the mouse VLPO(**a**) or PZ(**b**).

**Fig. S17 Effect of *Impdh2* knockout in PZ neurons on sleep architecture.**

**a.** Schematic of AAV-h*Syn*-mCherry delivery into the mouse PZ to generate Neu-cKO of *Impdh2*. **b.** Representative fluorescence images showing NeuN labeling and h*Syn*-mCherry expression in the mouse PZ. **c.** Western blot analysis confirming reduced IMPDH2 protein levels in the PZ of control and *Impdh2* PZ Neu-cKO mice (**n=3** mice per group). **d.** Hourly time spent in wake across the recording period (**n=9** mice per group). **e–g.** Quantification of wake parameters, including total wake duration (**e**), wake bout number (**f**), and mean wake bout duration (**g**). **h.** Hourly time spent in NREM sleep. **i–k.** Quantification of NREM sleep parameters, including total NREM duration (**i**), NREM bout number (**j**), and mean NREM bout duration (**k**). **l.** Hourly time spent in REM sleep. **m–o.** Quantification of REM sleep parameters, including total REM duration (**m**), REM bout number (**n**), and mean REM bout duration (**o**). **p–q.** Hourly relative EEG delta power during NREM sleep (**p**) and power spectral density of NREM sleep (**q**). Data are presented as mean ± SEM.

**Fig. S18 Effect of *Impdh2* knockout in the PZ on recovery sleep.**

**a.** Hourly time spent in NREM sleep during the recovery period following sleep deprivation (**n=7** mice per group). **b–d.** Quantification of NREM sleep parameters during recovery sleep, including total NREM duration (**b**), NREM bout number (**c**), and mean NREM bout duration (**d**). **e.** Mean wake bout duration across recovery sleep time stages. **f.** Hourly relative EEG delta power during NREM sleep in the recovery period. **g.** Power spectral density of slow-wave activity (SWA) during recovery NREM sleep. Data are presented as mean ± SEM.

**Fig. S19 Effect of *Impdh2* knockout in VLPO neurons on sleep architecture.**

a. Schematic illustrating AAV-h*Syn*-Cre injection into the mouse VLPO to generate Neu-cKO of *Impdh2*. **b.** Representative fluorescence images showing NeuN labeling and h*Syn*-mCherry expression in the mouse VLPO. **c.** Western blot analysis of IMPDH2 protein levels in the VLPO of control and *Impdh2* Neu-cKO mice (**n=3** mice per group). **d–g.** Wake parameters, including hourly time spent in wake (**d**), total wake duration (**e**), wake bout number (**f**), and mean wake bout duration (**g**). **h–k.** NREM sleep parameters, including hourly NREM sleep duration (**h**), total NREM duration (**i**), NREM bout number (**j**), and mean NREM bout duration (**k**) (**n=8** mice per group). **l–o.** REM sleep parameters, including hourly REM sleep duration (**l**), total REM duration (**m**), REM bout number (**n**), and mean REM bout duration (**o**). **p–q.** Hourly relative EEG delta power during NREM sleep (**p**) and power spectral density of NREM sleep (**q**). Data are presented as mean ± SEM.

**Fig. S20 Effect of *Impdh2* knockout in the VLPO neuron on recovery sleep.**

**a.** Hourly time spent in NREM sleep during the recovery period following sleep deprivation (**n=8** mice per group). **b–d.** Quantification of NREM sleep parameters during recovery sleep, including total NREM duration (**b**), NREM bout number (**c**), and mean NREM bout duration (**d**). **e.** Mean wake bout duration across recovery sleep time stages. **f.** Hourly relative EEG delta power during NREM sleep in the recovery period. **g.** Power spectral density of SWA during recovery NREM sleep. Data are presented as mean ± SEM.

**Fig. S21 Verification of Cre expression in the Right-side BF but not in the left-side BF. a.** Cre was not detected in the control-side BF. **b.** Cre was expressed in the Cre-side BF of the same brain slice. scale-bar: 200μm

**Fig. S22 Sleep-state–dependent neuronal activity in the basal forebrain is unchanged by *Impdh2* knockout.**

**a.** Schematic of the experimental design for bilateral recordings of BF neuronal activity using GCaMP6m fiber photometry in control and *Impdh2* knockout conditions. **b.** Validation of AAV injection and optical fiber placement in the BF. **c.** Representative GCaMP6m fluorescence signals recorded from the BF of control and *Impdh2* knockout sides. **d.** Quantification of average GCaMP6m signal peak amplitudes. **e.** Heatmaps (left), averaged time-course traces (middle), and cumulative signal comparisons (right) aligned to the NREM-to-wake transition. **f.** Heatmaps (left), averaged time-course traces (middle), and cumulative signal comparisons (right) aligned to the NREM-to-REM transition. Data are presented as mean ± SEM.

 **Fig. S23 K195 ubiquitination modulates IMPDH2-dependent sleep regulation.**

**a.** Quantification of NREM bout number following overexpression of IMPDH2-WT or IMPDH2-K195R in basal forebrain neurons. **b.** Mean NREM bout duration. **c.** Hourly time spent in wake across the recording period. **d–f.** Quantification of wake parameters, including total wake duration (**d**), wake bout number (**e**), and mean wake bout duration (**f**). **g.** Hourly time spent in REM sleep across the recording period. **h–j.** Quantification of REM sleep parameters, including total REM duration (**h**), REM bout number (**i**), and mean REM bout duration (**j**). **k–l.** Behavioral performance assessed by OFT and NOR. Sample size: **n=9** mice per group for panels **a–j**, and **n=10** mice per group for panels **k–l**. Data are presented as mean ± SEM.

**Fig. S24 K195 ubiquitination modulates IMPDH2-dependent sleep regulation via *Adora1*.**

**a.** Quantification of NREM bout number in BF Neu-cKO mice of *Adora1* overexpressing h*Syn*-mCherry, IMPDH2-WT or IMPDH2-K195R. **b.** Mean NREM bout duration. **c.** Hourly time spent in wake across the recording period. **d–f.** Quantification of wake parameters, including total wake duration (**d**), wake bout number (**e**), and mean wake bout duration (**f**). **g.** Hourly time spent in REM sleep across the recording period. **h–j.** Quantification of REM sleep parameters, including total REM duration (**h**), REM bout number (**i**), and mean REM bout duration (**j**), following overexpression of IMPDH2-WT or IMPDH2-K195R in BF neuron–specific *Adora1* knockout mice. **k–l.** Behavioral performance assessed by Open Field Test (OFT) and Novel Object Recognition (NOR). Sample size: **n=9** mice for *Adora1* cKO + mCherry, **n=8** mice for *Adora1* cKO + IMPDH2-WT and **n=9** mice for *Adora1* cKO + IMPDH2-K195R for panels **a–j**; **n=10** mice per group for panels **k–l**. Data are presented as mean ± SEM.

**Fig. S25 The effect of interfering sequence on endogenous *Impdh2* expression**

**a.** IS_436-455 and IS_456-475 did not change the expression of IMPDH2, **n=3** in each group.

**b.** IS_Fusion did not elevate the expression of IMPDH2. **n=3** in each group.

**Fig. S26 Confirmation of IS injection and *Btbd9* overexpression**

**a.** Fluorescence image of GFP, mCherry, and NeuN in the basal forebrain of WT mice.

**Fig. S27 Disruption of the interaction between *IMPDH2* and *BTBD9* alters sleep architecture.**

**a.** Hourly time spent in NREM sleep across the recording period. **b.** Power spectral density of NREM sleep. **c–d.** Quantification of NREM sleep parameters, including total NREM duration (**c**) and NREM bout number (**d**). **e.** Hourly time spent in NREM sleep under the indicated experimental conditions. **f–h.** Quantification of wake parameters, including total wake duration (**f**), wake bout number (**g**), and mean wake bout duration (**h**). **i.** Hourly time spent in REM sleep across the recording period. **j–l.** Quantification of REM sleep parameters, including total REM duration (**j**), REM bout number (**k**), and mean REM bout duration (**l**). ***n* = 10** per group for all panel above. Data are presented as mean ± SEM.

**Fig. S28 Effect of IS_Fusion expression on recovery sleep and behavioral performance.**

**a-c.** Quantification of NREM sleep parameters during recovery sleep, including total NREM duration (**a**), NREM bout number (**b**), and mean NREM bout duration (**c**)(**n=10** mice per group for EEG recordings). **d.** Hourly relative EEG delta power during NREM sleep in the recovery period. **e.** Cognitive performance assessed by the NOR test, shown as recognition index calculated from exploration bout number and exploration duration across the same four groups. **f.** OFT assessing locomotor activity, including total distance traveled, distance in the center, time spent in the center, and center–periphery transitions in four experimental groups: h*Syn*-mCherry + IS_Scramble (**n=12**), h*Syn*-*Btbd9* + IS_Scramble (**n=11**), h*Syn*-mCherry + IS_436–475 (**n=12**), and h*Syn*-*Btbd9* + IS_436–475 (**n=12**). Data are presented as mean ± SEM.

**Supplementary Table 1**. **Complete GTEx-derived eQTL dataset for BTBD9-associated variants across human tissues.** This table provide the full eQTL data of *BTBD9* that was used for association study with published GWAS study and PheWAS study with UKB data in **Fig. S1a.**

**Supplementary Table 2**. **Functional eQTL analysis of *BTBD9* sleep-related SNPs based on the GTEx dataset.** This data summarizes the GWAS studies included in this analysis, along with the full list of reported significant (lead) SNPs associated with sleep-related phenotypes. In addition to the genetic association results, the table provides key summary statistics for each variant, including SNP identifiers, genomic context, P-values, effect sizes (odds ratios or beta coefficients), and corresponding 95% confidence intervals. For functional interpretation, we further annotate variants with available expression quantitative trait loci (eQTL) information derived from the GTEx (v10) database, highlighting those SNPs with evidence of regulatory effects on *BTBD9* expression.

**Supplementary Table 3**. **Significant *BTBD9* SNPs associated with sleep phenotypes and their tissue-specific eQTL effects based on public GWAS datasets.** This table provides a comprehensive list of all reported BTBD9-associated positive variants with full eQTL information, serving as a complementary dataset for functional interpretation for **Supplementary Table 2**.

**Supplementary Table 4**. **Significant association between *BTBD9* SNPs and sleep traits in the UKB cohort.** Summary statistics of the association analysis between BTBD9 variants and daytime sleepiness in the UK Biobank participants (N = 404,522) under application 493597. Linear and binomial regression models were adjusted for age, sex, BMI, smoking, alcohol consumption, and Townsend Deprivation Index (TDI). Reported *P*-values were corrected for multiple testing using False Discovery Rate (FDR) and Bonferroni methods.

**Supplementary Table 5**. **Key reagents and resources.**

**Supplementary Table 6**. **Summary of statistical analyses for all figures in the manuscript.** All statistical methods, test statistics, and P values for data presented in both the main text and supplementary figures are reported in this table.

**Supplementary Table 7.** This file contains the original Western blot images corresponding to all figures included in the manuscript. Each entry is labeled according to figure number, marker, and target protein. Representative images used in the manuscript figures are derived from these raw datasets.
